# Dysregulation of sphingolipid and cholesterol homeostasis imposes oxidative stress in human spermatozoa

**DOI:** 10.1016/j.redox.2025.103669

**Published:** 2025-05-15

**Authors:** Steven Serafini, Cristian O'Flaherty

**Affiliations:** a Department of Medicine, Experimental Medicine Division, McGill University, Montréal, Québec, Canada; b Department of Surgery, Urology Division, McGill University, Montréal, Québec, Canada; c The Research Institute, McGill University Health Centre, Montréal, Québec, Canada; d Department of Anatomy and Cell Biology, McGill University, Montréal, Québec, Canada; e Department of Pharmacology and Therapeutics, McGill University, Montréal, Québec, Canada

**Keywords:** Human spermatozoa, Sperm capacitation, Lipid signalling, Cholesterol efflux, Redox signalling, Dyslipidemia

## Abstract

Infertility is a significant public health concern, affecting one in six couples globally, with male-factor infertility representing half of all cases. Obesity is an important health problem and is increasingly linked to poor reproductive outcomes as it induces metabolic disturbances and dyslipidemia, both of which impair male fertility. Dyslipidemia, characterized by abnormal lipid profiles, is associated with declining sperm quality and alters cholesterol and sphingolipid abundances in the seminal plasma. Sperm capacitation enables spermatozoa to acquire fertilizing competence by promoting cholesterol efflux from the plasma membrane, modifying lipid composition and membrane fluidity, and enhancing the ability to recognize and fertilize the oocyte. However, the intricate interplay between cholesterol efflux and capacitation-associated modifications in human spermatozoa remains poorly understood. Sphingolipids, including Ceramide (Cer) and sphingosine-1-phosphate (S1P), are critical regulators of cellular functions that play a significant role in male reproductive health. These bioactive lipids and cholesterol promote sperm capacitation, motility, and the acrosome reaction (AR). We hypothesize that dysregulated lipid homeostasis, as seen in cases of dyslipidemia, can impair capacitation by disrupting lipid-signalling pathways and promoting oxidative stress. Our results demonstrate that methyl-β-cyclodextrin (MβCD), which extracts cholesterol from the sperm plasma membrane, enhances a variety of capacitation-associated adaptations, including tyrosine phosphorylation, hyperactive motility, and AR. Moreover, sphingolipid signalling, through the S1PR1/3 receptors, is crucial in mediating these effects. MβCD-cholesterol extraction is also associated with nitric oxide (NO) and superoxide (O_2_^•-^) production, key signalling molecules involved in capacitation. However, dysregulated lipid metabolism, characterized by elevated cholesterol sulfate (Chol-SO_4_), Cer, and Sphingosine (Sph), impairs sperm capacitation through excessive oxidative stress and promotes oxidative damage. The findings suggest that disruptions in lipid metabolism resulting from conditions like obesity can interfere with sperm capacitation and fertilizing potential, providing new insights into male infertility mechanisms.

## Highlights

• Sphingolipid signalling aids methyl-β-cyclodextrin-induced sperm capacitation and acrosome reaction.
• PI3K-AKT-NOS pathway activation and cholesterol efflux support human sperm capacitation.
• Cholesterol efflux drives early extracellular superoxide production in human sperm capacitation.
• Blocking nSMase prevents cholesterol efflux and reduces tyrosine phosphorylation during sperm capacitation.
• Imbalanced sphingolipid and cholesterol metabolism impairs sperm capacitation and acrosome reaction.

## Graphical abstract

**Figure:**
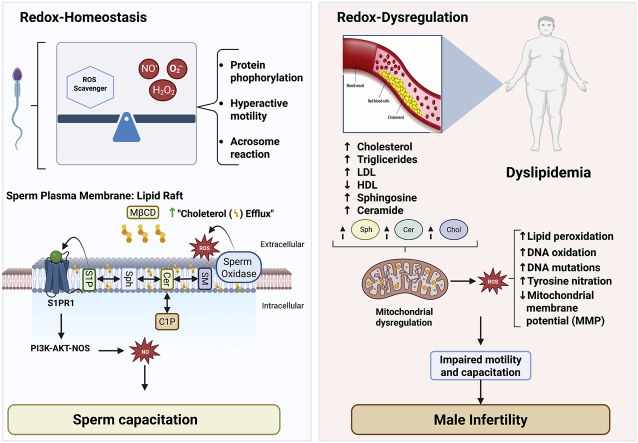

## 1 Introduction

Human infertility, a growing public health issue, affects one in six couples worldwide [1,2]. Infertility is a multifactorial condition with genetic, environmental, and lifestyle factors [3]. Male infertility contributes to approximately 50 % of all cases, with roughly 34 % being categorized as idiopathic [4]. Additionally, semen quality has declined globally over recent decades [5]. Moreover, obesity is a growing global health concern with significant impacts on reproductive health [6]. The obesity epidemic affects more than one-third of the worldwide population, 43 % of whom are men [7]. Obesity leads to adipose tissue dysfunction [8] and insulin resistance [9], which are associated with dyslipidemia, characterized by abnormal levels of triglycerides, decreased high-density lipoproteins (HDL), or an increase in low-density lipoproteins (LDL) in the bloodstream [10]. Dyslipidemia is a contributor to the global cardiovascular disease (CVD) burden [11,12] and accelerates the development of atherosclerosis [13], insulin resistance [14], and certain cancers [15]. Increasing evidence links dyslipidemia to rising rates of male infertility [16] by amplifying germ cell apoptosis [17,18], reducing the fertilization rate and implantation rate [[19], [20], [21], [22]], decreasing sperm counts [[23], [24], [25]], causing hormonal imbalance [26,27], and promotion of oxidative stress [16,[28], [29], [30], [31]]. While dyslipidemia has a complex impact on male reproductive function, its precise role in the fertilizing ability of the spermatozoon is still not well understood.

Sperm capacitation is a complex and time-sensitive process involving biochemical and biophysical alterations essential for acquiring hyperactive motility, allowing the spermatozoon to recognize and bind to the zona pellucida, initiate the acrosome reaction (AR), and successfully fertilize the oocyte. [32]. Sphingolipid signalling [33] and cholesterol efflux [[34], [35], [36], [37]] are important prerequisites for attaining fertilizing potential. A high-fat diet can result in cholesterol and sphingolipid imbalance in the semen [38], negatively affecting sperm capacitation.

Sphingolipids are bioactive lipids crucial in regulating cellular processes, including signal transduction, membrane structure, and cell motility [[39], [40], [41], [42]]. Among the members of sphingolipids, ceramide (Cer) and sphingosine 1-phosphate (S1P) have been well-studied for regulating cellular functions influencing diverse physiological [43,44] and pathological [[45], [46], [47]] processes. Sphingolipid metabolism is tightly controlled [48] with enzymes such as sphingomyelinases (SMase), ceramidases, sphingosine kinases, and S1P lyases, balancing the synthesis, degradation, and interconversion of sphingolipids [49]. This finely tuned metabolic network is crucial for maintaining cellular homeostasis, as dysregulation can lead to various diseases, including CVD [50], metabolic disorders [51], and neurodegenerative disorders [52]. Sphingolipids are critical for male reproductive health and constitute about 10–20 % of total membrane lipids [53]. The primary sphingolipids in seminal plasma and spermatozoa are Cer and sphingomyelin (SM) [38]. Sphingolipid metabolites influence spermatogenesis [54], preventing apoptosis in the testes [55], promoting sperm motility [56], capacitation [33], and AR [53,57], which are critical processes for successful fertilization.

Cholesterol is tightly regulated in the male reproductive tract, as lipid concentrations in blood serum do not correlate with seminal plasma [58]. The seminal plasma contains high cholesterol levels, approximately 250 μg/mL of cholesterol, predominantly found bound to lipoproteins and vesicles and are decapacitation factors [59]. A key change during capacitation is a reduction of the cholesterol: phospholipid ratio, resulting from cholesterol efflux from the plasma membrane to albumin, high-density lipoproteins, and MβCD in both *in vitro* [60] and *in vivo* [37,61]. This shift in membrane lipid composition significantly affects the membrane's biophysical properties, particularly its fluidity, which may influence protein function, impact ion channel and enzymatic activity, and increase protein tyrosine phosphorylation (P-Tyr) [34]. Methyl β-cyclodextrin (MβCD), a cyclic heptasaccharide consisting of β(1–4)-glucopyranose units, solely promotes cholesterol efflux [62]. MβCD drives the release of cholesterol from the mouse sperm plasma membrane in media devoid of albumin, leading to an increase in P-Tyr [34].

We hypothesized that cholesterol efflux activates specific signal-transduction cascades that regulate key processes during sperm capacitation, including P-Tyr, hyperactive motility, and AR. Dysregulation of sphingolipid and cholesterol homeostasis leads to impaired sperm fertilizing potential by increasing oxidative stress and damage. This study aims to explore the novel molecular mechanisms regulated by cholesterol efflux that promote human sperm capacitation and to examine how alterations in sphingolipid and cholesterol balance disrupt sperm function, thereby compromising fertilization potential through enhanced oxidative stress and cellular damage.

## 2 Materials and methods

### 2.1 Materials

Rabbit polyclonal anti-phospho-PI3K (P-PI3K) antibodies were purchased from Cell Signaling (**#**4228) (Beverly, MA, USA). Mouse monoclonal anti-P-Tyrosine (P-Tyr), clone 4G10 (#05–321**)**, nitrocellulose blotting membrane (GE10600004), Nω-Nitro-l-arginine methyl ester hydrochloride (l-NAME) (N5751), progesterone (P0130), *Pisum sativum* lectin conjugated with FITC (PSA-FITC) (L0770), Superoxide Dismutase (SOD) from bovine liver (S8160), and bovine serum albumin (BSA) (A9418), C6 ceramide (d18:1/6:0) (860506), sphingosine (d18:1) (860490), and cholesterol sulfate (Chol-SO_4_) (700016) were purchased from Millipore Sigma Canada (Oakville, ON, Canada). S1PR1/3 inhibitor (VPC 23019) (#4195) was purchased from Tocris Bioscience (Bristol, UK). BODIPY-cholesterol (HY-125746) was purchased from MedChem Express (Monmouth Junction, NJ, USA). Horseradish peroxidase-conjugated goat anti-mouse IgG (#115-035-062) and donkey anti-rabbit IgG (#711-035-152) antibodies were purchased from Jackson Laboratories (Bar Harbor, ME, USA). Percoll (#45-001-747), enhanced chemiluminescence (ECL) Western Blotting Substrate (PI32106), goat anti-mouse (H + L)(A-11001) and donkey anti-goat (H + L) antibodies conjugated with AlexaFluor 555 (A-32727) and AlexaFluor 488 (A-11001), SYTOX™ Blue (S34857), propidium iodide (PI) (P1304MP), Mitochondrial Superoxide Indicator (MitoSOX™) (M36008), JC-1 (T3168), and BODIPY™ 581/591 C11 (D3861) were all sourced from ThermoFisher Scientific (Markham, ON, Canada). 3-*O*-Methyl-Sphingomyelin (3-OMS) (ab141756), Anti-8-hydroxy-2′-deoxyguanosine-FITC conjugated (ab183393), and Anti-3-Nitrotyrosine (ab61392) antibodies were purchased from Abcam (Toronto, ON, Canada). DAF-2 diacetate (DAF-2DA) (#85165) was sourced from Cayman Chemicals (Ann Arbor, MI, USA). 6-(4-Methoxyphenyl)-2-methyl-3,7-dihydroimidazo[1,2-*a*]pyrazin-3(7H)-one-hydrochloride (MCLA, #87787), methyl-β-cyclodextrin (MβCD) (C4555), anti-α-Tubulin (α-Tub) antibody and all other chemicals used were of reagent grade and purchased from Sigma-Aldrich (Milwaukee, WI, USA). Human fetal cord serum samples were collected from the Cellular Therapy Laboratory at the Research Institute, McGill University Health Centre. Fetal cord ultrafiltrates (FCSu) were prepared using Amicon Ultra-4 filter devices (UFC8010D) with membranes having a 3 kDa exclusion limit (MilliporeSigma, Oakville, ON, Canada), following previously established procedures [63].

### 2.2 Subjects and sperm sample preparation

This study received approval from the Ethics Board of the McGill University Health Centre, and informed consent was obtained from all 15 participants. In every experiment, different donor samples were used to avoid bias. Semen samples were collected from healthy donors aged 18–30 years following 72 h of sexual abstinence. The samples were incubated at 37 °C for 30 min to facilitate liquefaction, then separated using a four-layer Percoll gradient (20 %, 40 %, 65 %, 95 %) to isolate highly motile sperm at the 95 % Percoll layer and the 65 %–95 % Percoll interface (sperm preparation devoid of many abnormal spermatozoa, round cells, or other cellular elements). Sperm motility was assessed with a Hamilton Thorne computer-assisted sperm analysis system using HTCASAII software V1.17 (Beverly, MA, USA). Only samples exhibiting progressive motility greater than 70 % were used. Sperm concentration was determined using an Improved Neubauer hemacytometer and adjusted to 50 × 10^6^ spermatozoa/mL with Biggers, Whitten, and Whittingham (BWW) medium, which contains 91.5 mM NaCl, 4.6 mM KCl, 1.7 mM CaCl_2_, 1.2 mM KH_2_PO_4_, 1.2 mM MgSO_4_, 5.6 mM d-glucose, 0.25 mM sodium pyruvate, 21.6 mM sodium lactate, and 20 mM HEPES at pH 7.95 [64]. The sperm samples were then incubated at 37 °C for 3.5 h with or without 10 % v/v fetal cord serum ultrafiltrate (FCSu), an established inducer of human sperm capacitation [[65], [66], [67]], or various concentrations (0.1–2.5 mM) of MβCD. FCSu induces similar capacitation-related changes as BSA/bicarbonate (e.g., increased protein tyrosine phosphorylation, hyperactivation, and responsiveness to AR inducers). Because BSA interferes with reactive oxygen species (ROS) measurements [63], we omitted BSA as an inducer of capacitation in some of our experiments. Sph and Cer were first resuspended in DMSO to form the stock solutions. After this, the working solutions of Sph and Cer were prepared in BWW and BSA with a final concentration of 0.12 mg/mL, which had been demonstrated in our lab to be lower than 3 mg/mL, required for inducing capacitation in human spermatozoa. Sperm capacitation was assessed by evaluating spermatozoa's P-Tyr levels and spermatozoa's ability to undergo progesterone-induced AR, well-established markers of human sperm capacitation [68]. We then determined the impact of MβCD-, sphingolipid-, and Chol–SO_4_–treatments on sperm viability, ROS production, and oxidative stress, as described below.

### 2.3 Sperm viability and motility determinations

Sperm viability was assessed using a modified hypo-osmotic swelling (HOS) test [69,70]. Following treatment, sperm samples were gently mixed with 150 μL of hypoosmotic solution (1.5 mM fructose and 1.5 mM sodium citrate) and incubated at 37 °C for 30 min. The samples were then placed on Superfrost Plus slides. Sperm viability was examined using a Leica DFC 450C microscope at 200 × magnification, with Leica Application Suite X (LASX) software (Version 1.1.0.12420, Leica Microsystems, Wetzlar, Germany). A total of 200 cells were analyzed in duplicate per sample, and only viable spermatozoa exhibiting varying degrees of tail curling or the presence of a droplet were considered.

Sperm motility was assessed using a computer-assisted semen analysis (CASA) [33]. Each sample tube received 3 mg/mL BSA, and the samples were thoroughly mixed and then loaded onto a Makler chamber. Motility was then evaluated using the HT-IVOS II CASA system (Hamilton Thorne, Beverly, MA, USA) set at 37 °C. At least 200 spermatozoa were analyzed to assess total, progressive, and hyperactivated motility. Total motility was calculated as the percentage of motile sperm relative to the total sperm count. Progressive motility was defined as the percentage of sperm exhibiting a time-average velocity (VAP) ≥ 25 μm/s and a straightness of trajectory (STR) ≥ 80 % [71]. Hyperactive motility was defined as the percentage of cells with a Curvilinear velocity (VCL) ≥ 150 mm/s AND linearity (LIN) ≤ 50 % AND ALH ≥7.0 mm [71].

### 2.4 Acrosome reaction (AR) determination

Following an incubation for 3.5 h at 37 °C with or without FCSu or MβCD, sperm samples were washed twice with fresh BWW by centrifugation at 600×*g* for 5 min to remove any proteins shed during sperm capacitation. The pellets were resuspended in BWW containing 10 mM progesterone, as described by Baldi et al. [72], and incubated for 30 min at 37 °C. After a second centrifugation at 600×*g* for 5 min, the supernatant was discarded, and the pellet was resuspended in 95 % ethanol. A 20 μL aliquot of ethanol-fixed sperm was placed onto Superfrost slides. Without drying, 20 μL of PSA-FITC (30 μg/mL) was added to the samples and incubated at 37 °C for 5 min. Following incubation, the slides were washed with distilled water, dried, and a drop of prolonged antifade-DAPI was applied. The slides were then covered with a coverslip. The percentage of spermatozoa with intact acrosomes (fluorescence present in the acrosome) and reacted acrosomes (absence of fluorescence in the acrosome) was determined by analyzing 200 spermatozoa per sample using a Zeiss LSM780 Laser Scanning Confocal Microscope (Opti-Tech Scientific, Montreal, QC, Canada) at 100 × magnification.

### 2.5 SDS-PAGE and immunoblotting

After 3.5 h of incubation, the sperm proteins were supplemented with a sample buffer containing 100 mM dithiothreitol and a cocktail of phosphatase inhibitors and boiled for 5 min at 100 °C. Then, samples were centrifuged at 21,000×*g* for 5 min at room temperature. Samples were loaded onto a 10 % polyacrylamide gel. Electrophoresis occurred for up to 1 h at a constant of 0.025 amps per gel. Following electrophoresis, sperm proteins were electrotransferred onto nitrocellulose membranes for 45 min at 100 V. Then, the membranes were blocked in 5 % skim milk in Tris-buffered saline containing 0.1 % v/v Tween 20 (TTBS) for 1 h. Incubation with primary antibodies occurred either at room temperature for 1 h for anti-P-Tyr antibody (1:10,000v/v) or overnight for anti-3-nitrotyrosine antibody (1:1,000v/v) (to assess tyrosine nitration) at 4 °C. After the membranes were washed with TTBS, they were incubated with their respective horse-radish peroxidase-conjugated goat anti-mouse or donkey anti-rabbit antibodies for 45 min at room temperature. The blots were imaged using an Amersham Imager 680 supplied by GE Healthcare (Montreal, QC, Canada). The positive immunoreactive protein bands were detected using chemiluminescence.

After immunoblotting, the nitrocellulose membranes were washed three times with deionized water, each lasting 10 min. The membranes were then treated with a silver stain solution (2 % w/v trisodium citrate, 0.8 % w/v FeSO4, and 0.2 % w/v AgNO3 in deionized water) as previously described [64]. The silver stain solution, containing silver nitrate, facilitates the binding of silver to amino acid side chains, particularly carboxyl and sulfhydryl groups, which makes it an effective method for assessing total protein content on the membrane. Once the staining signal reached its peak, the membrane was rinsed with deionized water and subsequently incubated with three drops of Farmer's reducer (0.005 % sodium carbonate, 0.15 % potassium hexacyanoferrate, 0.3 % thiosulfate) to intensify the stain. Loading controls were also confirmed by incubating the same membrane with anti α-Tubulin (1:10,000) antibody. The membrane was then imaged using the Amersham Imager 680.

The relative intensities of the protein bands (e.g., for P-Tyr the 105 kDa and 85 kDa bands) for each sample were determined using FIJI Image J software version 2.1.0/1.53c (National Institutes of Health, Stapleton, NY, USA) and normalized to that of the loading control obtained with silver stain (105 kDa and 65 kDa bands). A second normalization of the protein bands of each sample was done by dividing by that of the non-capacitated control sample. The protein band relative intensity was expressed as mean ± standard error.

### 2.6 Cholesterol efflux assay

BODIPY-cholesterol stock solution was reconstituted in DMSO to a stock concentration of 1 mM. We then pre-treated the spermatozoon with BODIPY-cholesterol at a final concentration of 0.4 μM for 30 min at 37 °C. Samples were centrifuged at 650×*g* over 20 % Percoll, and cell pellets were recovered. Sperm samples were resuspended in BWW media a final concentration of 30 × 10^6^/mL and added to the appropriate samples treated with or without FCSu (10 % v/v), BSA (3 mg/mL)/HCO_3_^−^ (25 mM), MβCD (0.5 mM), and 3-OMS (10 μM). Following incubation for 3.5 h at 37 °C, the samples were smeared onto Superfrost slides and then dried at room temperature. Samples were fixed using 4 % paraformaldehyde. Samples were rehydrated with PBS + Triton (0.1 % v/v) (PBS-T) for 20 min. Samples were washed, and a Prolong Antifade with DAPI (Fisher Scientific, Ottawa, ON, Canada) was added before coverslip application. The negative control was done by incubating spermatozoa without the fluorescent probe. The Zeiss LSM780 Laser Scanning Confocal Microscope was used at a magnification of 40 × , with the same exposure times for each sample. FIJI ImageJ software was used for background fluorescence subtraction and quantification of the average relative fluorescence intensity (RFI) of 200 spermatozoa per sample.

### 2.7 Determination of nitric oxide (NO) in spermatozoa by flow cytometry

NO levels in spermatozoa were quantified by flow cytometry using the diaminofluorescein-2 diacetate (DAF-2-DA, a sensitive and specific fluorescent probe for intracellular NO) [33,63]. Highly motile percoll-selected spermatozoa were diluted to 300 × 10^6^ spermatozoa/mL with 95 % Percoll to maintain the low viscosity and osmolality environment. Cells were incubated with 10 μM DAF-2DA for 30 min at 37 °C to load the fluorescent probe into the cells [63]. The sample tubes were kept in the dark during incubation. Afterward, the samples were further diluted with BWW to a final concentration of 50 × 10^6^ spermatozoa/mL and treated with or without FCSu (10 % v/v) or MβCD (0.5 mM), along with S1PR1/3 inhibitor (40 μM of VPC 23019) and incubated for an additional 3.5 h at 37 °C. Following treatment, spermatozoa were incubated with Sytox Blue (0.2 μM) to assess cell viability. The spermatozoa were washed, resuspended in 1000 μL of HEPES Buffered Saline (HBS), and analyzed by flow cytometry. Fluorescence signals from labelled spermatozoa were analyzed using a Becton Dickinson FACSCanto II flow cytometer (Becton Dickinson, San Jose, CA) equipped with a 488-nm argon laser. Nonsperm-specific events were excluded, and at least 10,000 spermatozoa were analyzed for each condition, with a flow rate of <100 cells/second. The percentage of DAF-2DA^+^ and Sytox-blue^+^ sperm cells and the mean fluorescence intensities were calculated and analyzed using FlowJo software (version 7.2.2, FlowJo, Ashland, OR).

### 2.8 Determination of superoxide anion (O_2_^•-^) production by chemiluminescence

Extracellular O_2_^•-^ as measured using MCLA-amplified luminescence on a TECAN M200 Pro Multimode plate reader (Männedorf, ZH, Switzerland) at 5-min intervals in integration mode (output summed for 10 s) at 37 °C, with constant sample mixing, as previously described [73]. Percoll-washed spermatozoa, at a concentration of 30 × 10^6^ cells/mL, were loaded onto a NunclonTM Delta Surface plate with BWW media, with or without FCSu and MβCD. Chemiluminescence was measured immediately after the addition of 20 μM MCLA, a highly sensitive, cell-impermeable probe. To ensure accurate results, each sample was run alongside a control sample supplemented with SOD1 (bovine Cu/Zn Superoxide Dismutase 1), allowing us to isolate the SOD-inhibitable (SOD-inh) signal. Appropriate blanks, consisting of incubation medium with or without SOD1, were also tested in parallel to account for any potential interference from the medium or substances being tested.

### 2.9 Sperm DNA oxidation

DNA oxidation in sperm treated with high cholesterol or high sphingolipid content was assessed by the level of 8-hydroxy 2-deoxyguanosine (8-OHdG) in cells treated with or without FCSu, MβCD, varying concentrations of Chol-SO_4_, and low or high doses of Sph and Cer for 3.5 h at 37 °C. Following treatments, spermatozoa were washed and incubated with 2 mM DTT in Phosphate-buffered saline (PBS) for 45 min at 37 °C to facilitate chromatin decondensation. After washing, the cells were incubated with anti-8OHdG FITC-conjugated antibody (1:1000) in 0.1 % Triton X-100 and 0.1 % sodium citrate (pH 7.4) for 30 min at 37 °C, protected from light. The samples were washed and stained with propidium iodide (PI) to quantify the percentages of FITC-positive cells out of the PI-stained cells with the 488 nm argon laser using the MACSQuant Analyzer flow cytometry.

### 2.10 Mitochondrial membrane potential

Per a previously established protocol [74], mitochondrial membrane potential (MMP) was assessed using the cationic carbocyanine dye JC-1. Spermatozoa were treated with or without FCSu, MβCD, varying concentrations of Chol-SO_4_, and low or high doses of Sph and Cer for 3.5 h at 37 °C. Samples were stained with 2 μM JC-1 for 15 min at 37 °C in the dark. Afterward, the cells were washed and stained with 0.2 μM Sytox Blue to exclude dead cells during flow cytometry analysis. At high mitochondrial membrane potential (MMP), JC-1 forms J-aggregates within the mitochondria, emitting red fluorescence detected by the B2 channel of the 488 nm argon laser. In a low MMP state, JC-1 remains in its monomeric form, emitting green fluorescence in the B1 channel. The results were expressed as the ratio of red to green fluorescence intensity, with a decrease in this ratio indicating mitochondrial depolarization.

### 2.11 Lipid peroxidation determination

Lipid peroxidation was determined using a BODIPY 581/591 C11 probe [75,76]. Briefly, spermatozoa were treated with or without FCSu, MβCD, the varying concentrations of Chol-SO_4_, and low or high doses of Sph and Cer for 3.5 h at 37 °C. Samples were stained with 5 μM BODIPY 581/591 C11 for 30 min at 37 °C in the dark. A positive control was prepared under the same conditions with 40 μM ferrous sulfate (FeSO_4_). Afterward, the cells were washed and stained with 0.2 μM Sytox Blue to exclude dead cells during analysis. A minimum of 10,000 events was analyzed for each sample using a MACSQuant Analyzer flow cytometer. Data are presented as the percentages of cells having a positive BODIPY C11 signal.

### 2.12 Determination of mitochondrial superoxide production

Mitochondrial O_2_^•-^ production was assessed using MitoSOX, a lipid-soluble cation that selectively targets mitochondria and is oxidized by O_2_^•-^ and fluoresces red upon binding to nucleic acid [77]. Spermatozoa were treated with or without FCSu, MβCD, varying concentrations of Chol-SO_4_, and low or high doses of Sph and Cer for 3.5 h at 37 °C. Samples were stained with 2 μM of MitoSOX and 0.2 μM of Sytox Blue for 15 min at 37 °C. Spermatozoa were washed and resuspended in HBS and analyzed by flow cytometry. The positive control for MitoSOX labelling was prepared by incubating a sperm aliquot with 40 μM of Antimycin A for 3.5 h at 37 °C. Data were analyzed as a percentage of cells producing mitochondrial O_2_^•−^.

### 2.13 Statistical analysis

All data are presented as mean ± SEM, with statistical differences between groups assessed using ANOVA followed by Tukey's test, conducted with GraphPad Prism 10 (GraphPad Software, Inc., San Diego, CA, USA). The normality of the data and homogeneity of variances were evaluated using the Shapiro-Wilk and Levene's tests, respectively. A p-value of ≤0.05 was considered statistically significant.

## 3 Results

### 3.1 MβCD-induced capacitation-related changes are inhibited with S1PR1/3 inhibitor VPC 23019

**Fig. 1:**
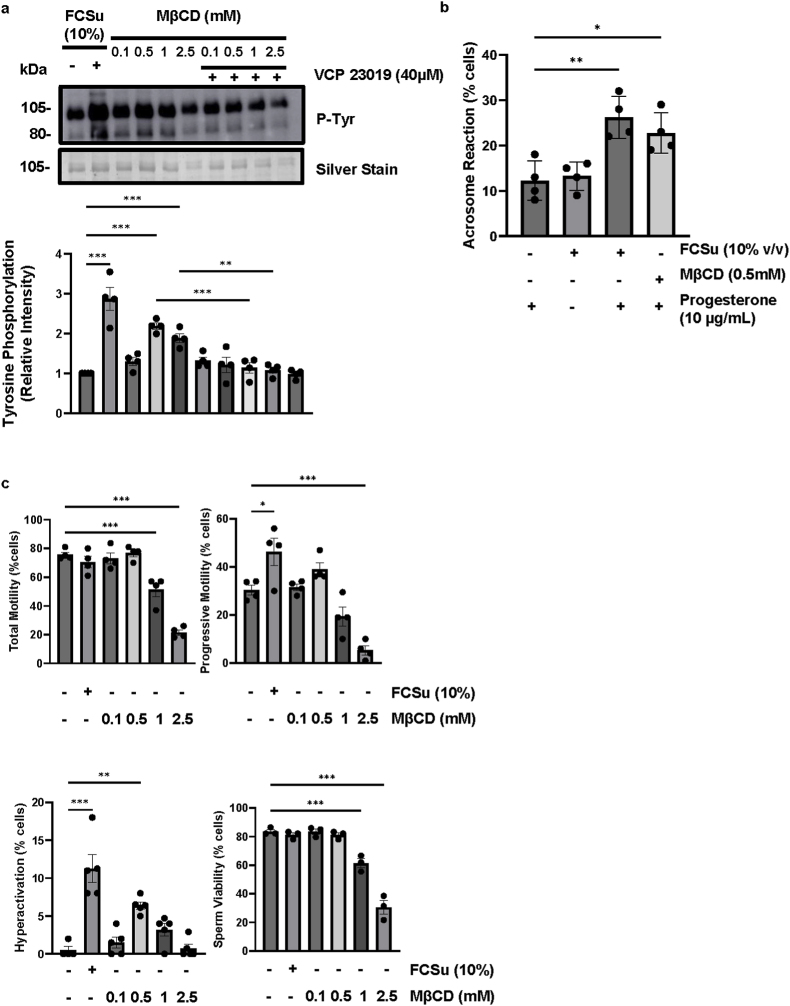
**MβCD-induced sperm capacitation is impaired by inhibiting S1PR1.** (**a**) Phospho-tyrosine (P-Tyr) intensity of the 80- and 105-kDa protein bands in spermatozoa of non-treated (−) or treated (+) with Fetal cord serum ultrafiltrate (FCSu 10 % v/v) or varying doses of methyl beta-cyclodextrin (MβCD) with or without 40 μM VPC 23019 (S1PR1/3 inhibitor) for 3.5h at 37 °C. P-Tyr at 0.5 mM of P-Tyr was mitigated when treated with VPC 23019. Each lane was normalized to its silver-stain optical density value for signal quantification. (**b**) Progesterone-induced acrosome reaction (AR) in spermatozoa pre-treated with or without the capacitation inducer FCSu (10 % v/v) or 0.5 mM MβCD for 3.5h at 37 °C. Following this treatment, sperm samples were either left untreated or exposed to 10 μM progesterone to induce the acrosome reaction. After incubation, the AR was assessed by measuring the percentage of spermatozoa without intact acrosomes, using fluorescence microscopy and PSA (Pisum sativum agglutinin) staining. Data represent the percentage of spermatozoa exhibiting the AR under each condition. MβCD-treated samples have an increased percentage of spermatozoa that underwent the induced AR compared to non-treated samples. Sperm samples not treated with progesterone showed no significant change in acrosome exocytosis compared to untreated controls. (**c**) Spermatozoa treated with MβCD for 3.5h at 37 °C were assessed for their motility and viability. 0.5 mM demonstrated an increase in hyperactive motility without impairing viability. The data represent sperm samples from four different healthy donors (n = 4). Statistical analysis was performed using ANOVA followed by Tukey's test. Significant differences are indicated as ∗p ≤ 0.05, ∗∗p ≤ 0.01, and ∗∗∗p ≤ 0.001.

The S1PR1/3 inhibitor VPC 23019 impairs sperm capacitation by disrupting the S1P signalling in human spermatozoa [33]. In our present study, P-Tyr levels (a marker of sperm capacitation) were increased by 0.5 mM MβCD. The MβCD-dependent increase of P-Tyr levels was impaired by treating spermatozoa with 40 μM VPC 23019 (Fig. 1a). MβCD also increased the progesterone-induced AR in live spermatozoa (Fig. 1b) compared to those observed in non-treated controls (without FCSu). In addition, FCSu alone did not promote AR in non-treated spermatozoa. Moreover, 0.5 mM of MβCD had no impact on total and progressive motility or viability but promoted an increase in hyperactivated motility (a distinctive motility characterized by non-linear sperm movement with a high amplitude flagellar beating that is observed in capacitated spermatozoa) compared to non-treated spermatozoa (Fig. 1c). These results demonstrate that MβCD-mediated cholesterol efflux renders the acquisition of several capacitation-associated modifications *in vitro.* Further experimentation was performed using 0.5 mM of MβCD.

### 3.2 MβCD promotes capacitation by engaging with the S1PR1 signalling cascade, promoting NO production

**Fig. 2:**
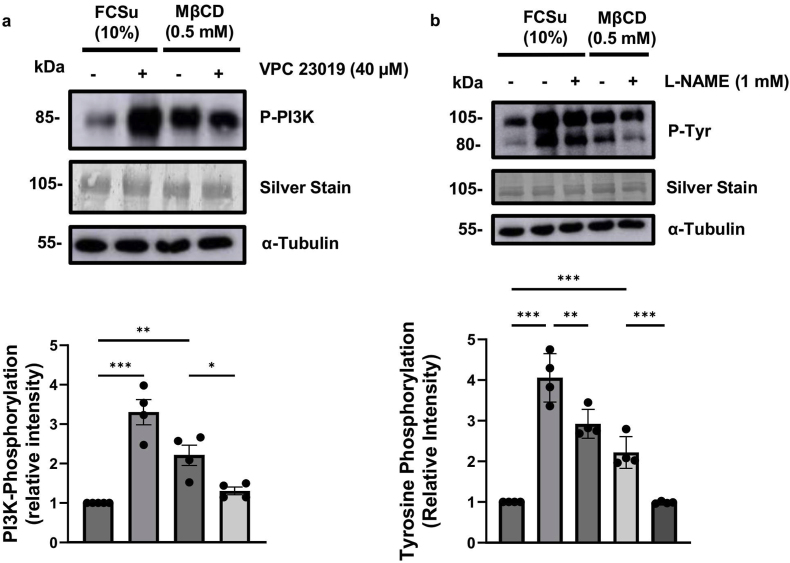
**MβCD increases PI3K phosphorylation, while inhibition of S1PR or NOS impairs MβCD-induced tyrosine phosphorylation.** Spermatozoa were incubated for 3.5 h at 37 °C under various conditions: untreated (−), treated with 10 % v/v fetal cord serum ultrafiltrate (FCSu), treated with 0.5 mM methyl-β-cyclodextrin (MβCD), and treated with MβCD in combination with (**a**) 40 μM VPC 23019 (S1PR1/3 inhibitor) or (**b**) l-NAME (Nitric Oxide Synthase inhibitor). (**a**) Immunoblotting analysis revealed that VPC 23019 at 40 μM decreased PI3K phosphorylation (P-PI3K) in spermatozoa incubated with 0.5 mM of MβCD. (**b**) Immunoblotting showed that l-NAME treatment decreased tyrosine phosphorylation (P-Tyr) in sperm samples treated with FCSu and MβCD. The signal intensity for each lane was normalized to the silver-stain optical density value for accurate quantification. α-Tubulin loading controls were performed for direct comparison with the silver stain loading controls. The data represent sperm samples from four different healthy donors (n = 4). Statistical analysis was performed using ANOVA with Tukey's test: ∗p ≤ 0.05, ∗∗p ≤ 0.01, and ∗∗∗p ≤ 0.001.

The PI3K pathway plays a crucial role in the capacitation process in humans [78]. In previous studies, we demonstrated that S1PR1-mediated signalling promotes capacitation-related modifications by activating the PI3K-AKT pathway [33]. We followed up on our previous results [33] to determine whether cholesterol efflux regulates S1PR1**-**mediated signalling to promote PI3K phosphorylation by incubating spermatozoa with or without FCSu or MβCD in the presence or absence of 40 μM of VPC 23019. We observed that both FCSu and MβCD increased P-PI3K levels compared to non-treated controls and those treated with VPC 23019 (Fig. 2a). This suggests that MβCD-mediated cholesterol efflux may modulate the activation of S1P signalling, facilitating capacitation-associated modifications and promoting cross-talk that activates the PI3K-AKT pathway via sphingolipid signalling.

**Fig. 3:**
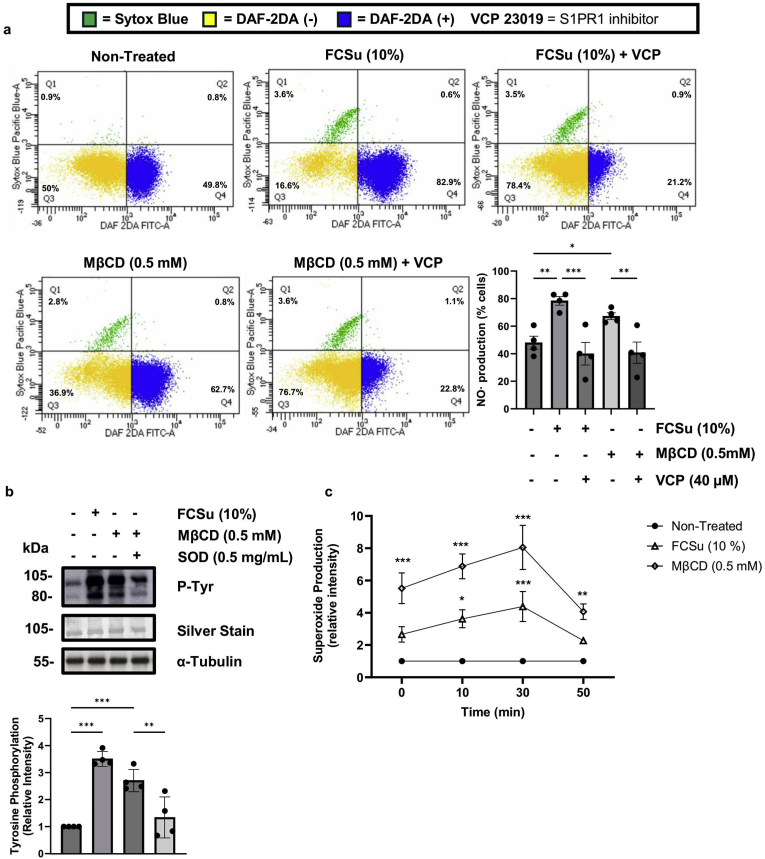
**MβCD promotes nitric oxide (NO) and superoxide (O_2_^•-^) production in human spermatozoa.** Spermatozoa were incubated for 3.5 h at 37 °C under various conditions: with (+) or without (−) 10 %v/v Fetal cord serum ultrafiltrate (FCSu) and 0.5 mM methyl β-cyclodextrin (MβCD) 40 μM VPC 23019 (S1PR1/3 inhibitor) and 0.5 mg/mL superoxide dismutase (SOD1) to assess various parameters. Intracellular NO levels were measured using DAF-2DA fluorescence, while sperm viability was evaluated using Sytox Blue fluorescence. Tyrosine phosphorylation (P-Tyr) was assessed by immunoblotting, and extracellular superoxide anion (O_2_^•-^) production was quantified using MCLA chemiluminescence. (**a**) Scatter plot depicting viable spermatozoa (Sytox Blue^−^) categorized based on nitric oxide (NO) production, as determined by DAF-2DA fluorescence. Spermatozoa were classified into DAF-2DA^+^ (blue, Q4), indicating NO production and DAF-2DA^−^ (yellow, Q3) for non–NO–producing cells. (**b**) Immunoblot analysis of P-Tyr in sperm samples incubated with sphingosine (Sph), ceramide (Cer), and 0.5 mg/mL SOD1. Spermatozoa were treated in BWW medium with or without 10 % v/v FCSu (-Δ-), with or without 0.5 mM MβCD (-◊-), and with or without 0.5 mg/mL SOD1. Chemiluminescence was measured following the addition of 20 μM MCLA, and the data show SOD-inhibitory chemiluminescence relative intensity, standardized to non-treated controls. α-Tubulin loading controls were performed for direct comparison with the silver stain loading controls. Data represent sperm samples from 4 different healthy donors (n = 4). Statistical analysis was performed using ANOVA with Tukey's test: ∗p ≤ 0.05, ∗∗p ≤ 0.01, and ∗∗∗p ≤ 0.001.

Phosphorylation of PI3K activates AKT, a kinase that phosphorylates several substrates, including nitric oxide synthase (NOS), an enzyme present in spermatozoa [79,80]. NOS is essential for human sperm capacitation [81,82]. Thus, we studied whether MβCD-cholesterol efflux can promote NOS activation. FCSu- or MβCD-treated spermatozoa were incubated in the presence or absence of 1 mM of l-NAME, a cell-permeable inhibitor of NOS that has been shown to prevent human sperm capacitation [73,[83], [84], [85], [86]]. P-Tyr levels decreased in FCSu- or MβCD-treated spermatozoa compared to controls without l-NAME (Fig. 2b). We also assessed the number of viable (Sytox blue^—^) spermatozoa that produced NO (DAF-2DA^+^) to determine whether cholesterol efflux cross-talk with S1PR1 signalling promotes NO production. Thus, we incubated spermatozoa with FCSu or MβCD with or without VPC 23019. We observed an increase in NO in FCSu-, MβCD-treated samples compared to the non-treated condition, as depicted by the greater number of viable cells in the DAF-2DA^+^ quadrant (blue, Q4) compared to the number of viable cells in the DAF-2DA^—^ quadrant (yellow, Q3) (Fig. 3a). NO production during capacitation-inducing conditions was reduced by S1PR1/3 inhibitor VPC 23019 (Fig. 3a). This result shows that MβCD-induced capacitation is associated with S1P–S1PR1 signalling to yield intracellular production of NO, a major player in human sperm capacitation.

### 3.3 MβCD-induced capacitation is associated with superoxide (O_2_^•-^) production

We further evaluated MβCD-induced capacitation on redox signalling by assessing whether it promotes extracellular O_2_^•-^ production. Spermatozoa treated with 0.5 mM of MβCD in the presence of O_2_^•-^ scavenger SOD1 at 0.5 mg/mL displayed lower levels of P-Tyr (Fig. 3b). Moreover, quantification studies demonstrated that both FCSu- and MβCD-treated samples, but MβCD to a far greater extent, are associated with increased extracellular O_2_^•-^ production (Fig. 3c). The time course of O_2_^•-^ production in Fig. 3c is similar to findings previously reported [73,87]. Thus, cholesterol efflux is involved in the production of O_2_^•-^ at the outer leaflet level during human sperm capacitation.

### 3.4 Neutral sphingomyelinase (nSMase) inhibition impairs MβCD-induced capacitation

**Fig. 4:**
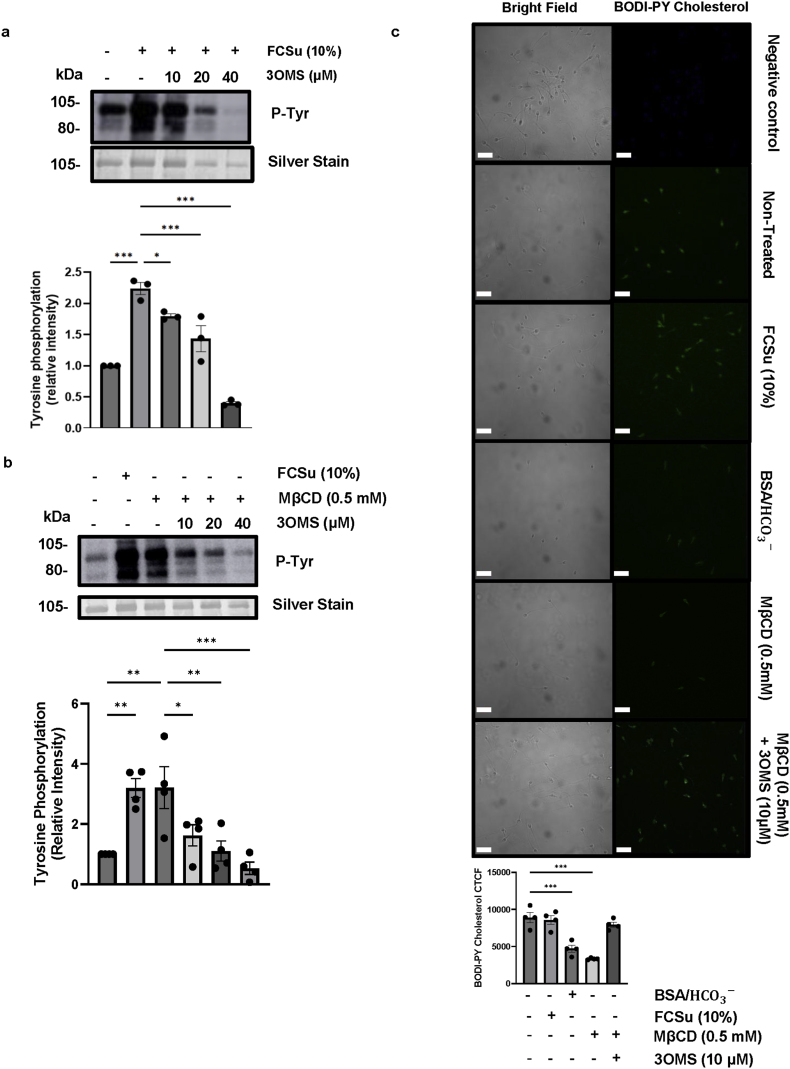
**MβCD cholesterol efflux and capacitation are reversed by nSMase inhibition.** Spermatozoa were capacitated in the presence of 10 % v/v fetal cord serum ultrafiltrate (FCSu) and 0.5 mM methyl-β-cyclodextrin (MβCD) for 3.5 h at 37 °C, with (+) or without (−) treatment with 3-OMS (a neutral sphingomyelinase [nSMase] inhibitor). (**a & b**) Immunoblotting analysis revealed a dose-dependent decrease in tyrosine phosphorylation (P-Tyr) with 3-OMS treatment in both FCSu- and MβCD-treated sperm. Signal intensity was quantified by normalizing each lane to its silver-stain optical density. (**c**) Immunocytochemistry showed no significant change in cholesterol labelling with BODIPY-cholesterol between untreated and FCSu-treated spermatozoa. However, bovine serum albumin (BSA) and MβCD treatments reduced cholesterol labelling, and 3-OMS restored labelling in MβCD-treated sperm (scale bar = 10 μm). Data represent sperm samples from 4 different healthy donors (n = 4). Statistical analysis was performed using ANOVA with Tukey's test: ∗p ≤ 0.05, ∗∗p ≤ 0.01, and ∗∗∗p ≤ 0.001.

**Supplementary Fig. S1:**
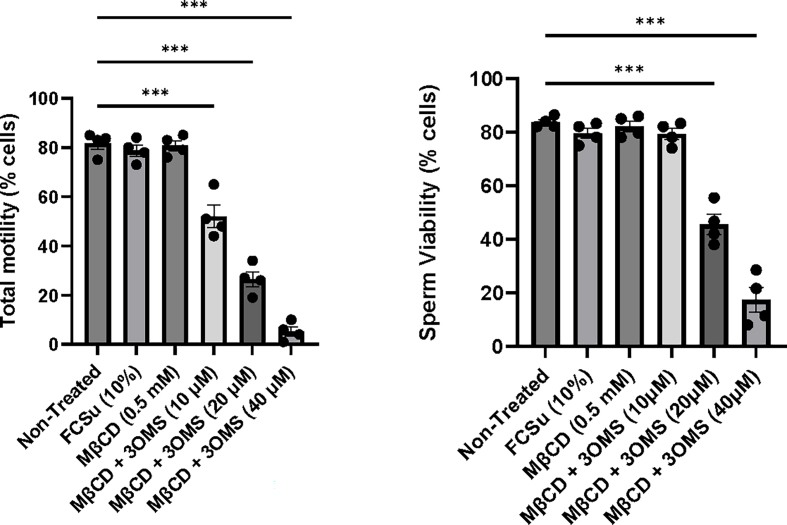
Total Motility and viability of nSMase i**nhibition.** Spermatozoa were incubated for 3.5 h at 37 °C, either untreated (Non-Treated) or treated with fetal cord serum ultrafiltrate (FCSu 10 % v/v) and increasing concentrations of 3-*O*-Methyl-Sphingomyelin (3-OMS, nSMase inhibitor). (**a**) Sperm motility was determined using computer-assisted semen analysis, with values expressed as a percentage of motile spermatozoa. (**b**) Sperm viability was assessed using the hypo-osmotic swelling (HOS) test, with results expressed as a percentage of live spermatozoa. At least 200 spermatozoa were recorded for each analysis. Data represent sperm samples from 4 different healthy donors (n = 4). Statistical analysis was performed using ANOVA with Tukey's test: ∗p ≤ 0.05, ∗∗p ≤ 0.01, and ∗∗∗p ≤ 0.001.

Previously, it was reported that SM influences the rate of human sperm acrosome reaction by regulating sterol loss [88]. However, the effects of cholesterol retention by inhibiting nSMase on sperm capacitation were not assessed. We examined the impact of inhibiting nSMase using different doses of 3-OMS on spermatozoa, both with and without treatment with FCSu or MβCD. FCSu- and MβCD-treated spermatozoa showed a dose-dependent decrease in P-Tyr with 3-OMS inhibition (Fig. 4a and b). Specifically, 10 μM of 3-OMS was the only concentration that did not impair viability, while all three concentrations impaired motility (Supplementary Fig. S1). We then evaluated if the decrease in P-Tyr and loss of motility were mediated by cholesterol retention. Cholesterol efflux was evaluated using BODIPY-cholesterol in spermatozoa treated with or without FCSu, BSA, MβCD, and MβCD with 10 μM of 3-OMS. BSA and MβCD treatment was associated with a significant decrease in BODIPY-cholesterol labelling, and the inclusion of 10 μM 3-OMS in the MβCD-treated spermatozoa resulted in the retention of BODIPY-cholesterol labelling (Fig. 4c). Moreover, FCSu-treated spermatozoa did not have a detectable increase in cholesterol efflux, confirming previous observations [83]. Thus, the inhibition of nSMase results in cholesterol retention, impairing key physiological changes necessary for sperm fertilizing ability.

### 3.5 Increased concentrations of Chol-SO_4_, Sph, or Cer impair P-Tyr, motility and viability

**Fig. 5:**
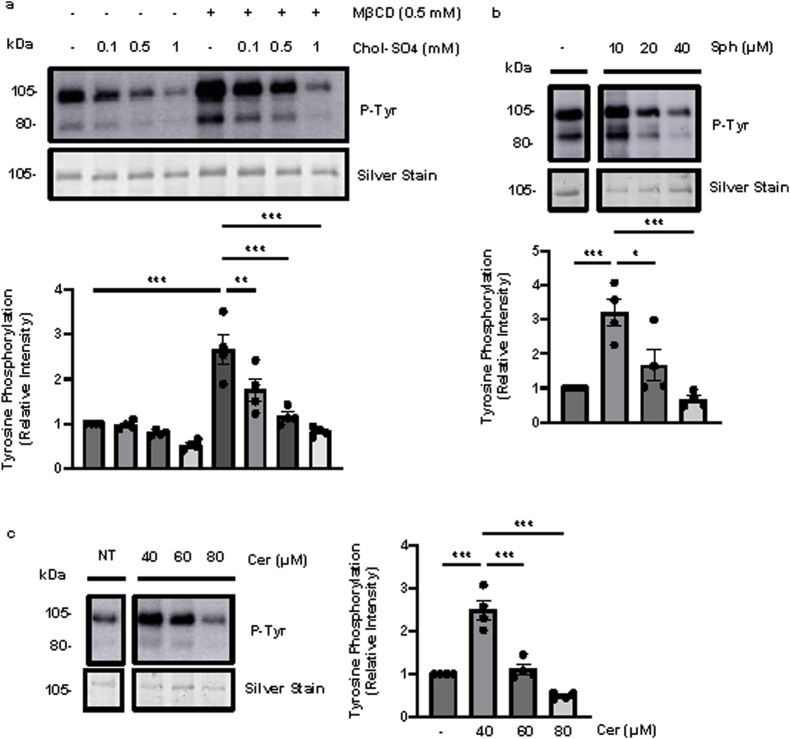
**High levels of cholesterol, Sphingosine, and Ceramide impair capacitation.** Spermatozoa were incubated for 3.5 h at 37 °C, with (+) or without (−) (**a**) 0.5 mM methyl-β-cyclodextrin (MβCD) and increasing concentrations of cholesterol-sulfate (Chol-SO_4_), (**b**) 10–40 μM sphingosine (Sph), or (**c**) 40–80 μM ceramide (Cer), to assess tyrosine phosphorylation (P-Tyr) fluorescence. Immunoblot analysis revealed a dose-dependent decrease in P-Tyr in human spermatozoa treated with increasing concentrations of Chol-SO_4_, Sph, and Cer compared to controls (MβCD alone, Sph 10 μM, and Cer 40 μM, respectively). All lines correspond to the same blot for sphingosine or ceramide treatments. Signal quantification was performed by normalizing each lane to its silver-stain optical density. Data represent sperm samples from 4 different healthy donors (n = 4). Statistical analysis was performed using ANOVA with Tukey's test: ∗p ≤ 0.05, ∗∗p ≤ 0.01, and ∗∗∗p ≤ 0.001.

**Supplementary Fig. S2:**
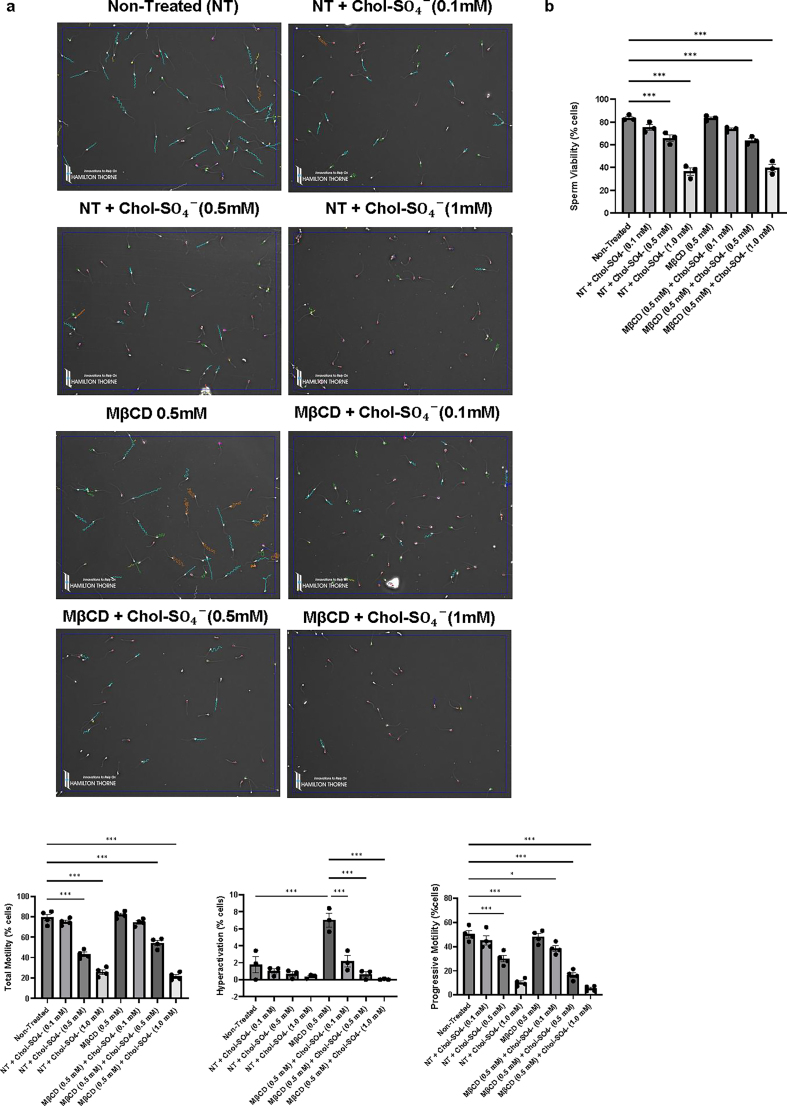
Motility and viability of sperm with a dose-dependent increase of cholesterol-sulfate (Chol-SO_4_). Spermatozoa were incubated for 3.5 h at 37 °C, without any treatment (Non-Treated), or with methyl β-cyclodextrin (MβCD 0.5 mM) and increasing concentrations of Chol-SO_4_. (**a**) Motility was determined using computer-assisted semen analysis. The blue tracks represent progressive motility, the orange tracks are hyperactive, and the green tracks are slow motility. Values for total, hyperactive, and progressive motility are expressed as a percentage. (**b**) Viability was assessed using the hypo-osmotic swelling (HOS) test. The values are expressed as a percentage of live spermatozoa. At least 200 spermatozoa were recorded for analysis. The results represent sperm samples from 4 different healthy donors (n = 4, ANOVA and Tukey test: ∗p ≤ 0.05; ∗∗p ≤ 0.01; and ∗∗∗p ≤ 0.001).

Previously, it was reported that in mouse spermatozoa, MβCD-mediated increase in P-Tyr was reversed by adding Chol-SO_4_ [34]. Here, we demonstrated that MβCD-driven P-Tyr (Fig. 5a), hyperactivation (Supplementary Fig. S2a), and viability (Supplementary Fig. S2b) were impaired by the dose-dependent increase in Chol-SO_4_ in human spermatozoa. Hence, a disproportionate amount of Chol-SO_4_ can impair capacitation-associated modifications.

**Supplementary Fig. S3:**
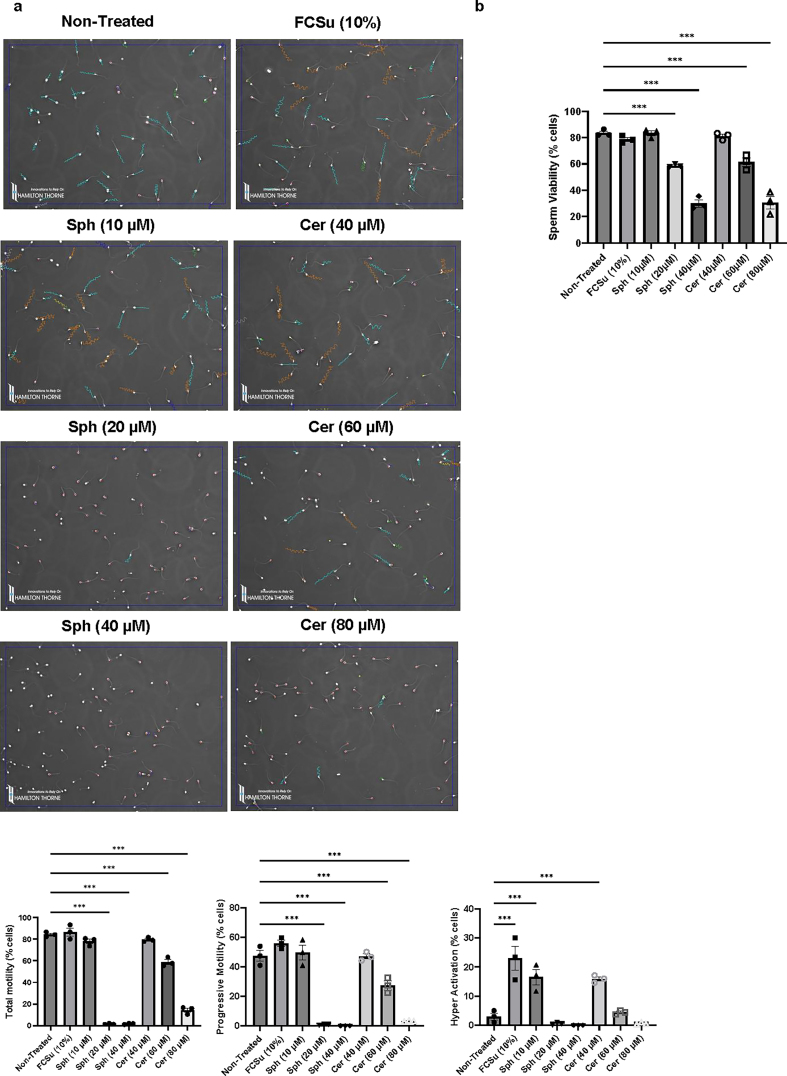
Motility and viability of sperm with a dose-dependent increase of Sphingosine (Sph) or Ceram**ide (Cer).** Spermatozoa were incubated for 3.5 h at 37 °C, either untreated (Non-Treated) or treated with fetal cord serum ultrafiltrate (FCSu 10 % v/v) and increasing concentrations of Sph or Cer. (**a**) Sperm motility was assessed using computer-assisted semen analysis. Blue tracks represent progressive motility, orange tracks indicate hyperactive motility, and green tracks correspond to slow motility. Values for total motility, hyperactive motility, and progressive motility are expressed as percentages. (**b**) Sperm viability was evaluated using the hypo-osmotic swelling (HOS) test, with results expressed as a percentage of live spermatozoa. At least 200 spermatozoa were recorded for analysis. Data represent sperm samples from 4 different healthy donors (n = 4). Statistical analysis was performed using ANOVA with Tukey's test: ∗p ≤ 0.05, ∗∗p ≤ 0.01, and ∗∗∗p ≤ 0.001**.**

Moreover, as we previously demonstrated [33], Sph at 10 μM and Cer at 40 μM can increase P-Tyr (Fig. 5b & c). However, elevated dosages of Sph (20 and 40 μM) and Cer (60 and 80 μM) impaired P-Tyr (Fig. 5b & c), all sperm motility parameters (Supplementary Fig. S3a), and sperm viability (Supplementary Fig. S3b). Thus, elevated levels of the Sph and Cer impair the functioning of spermatozoa.

### 3.6 Increasing concentrations of Chol-SO_4_ promote oxidative stress

**Fig. 6:**
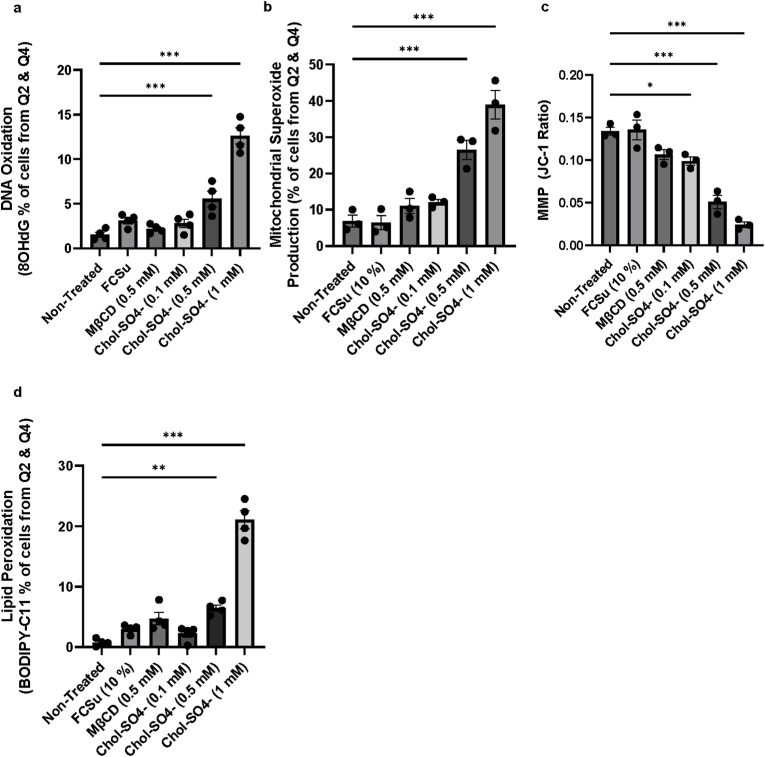
**High levels of cholesterol invoke oxidative stress and damage.** Spermatozoa were incubated for 3.5 h at 37 °C with or without treatment (Non-Treated) with fetal cord serum ultrafiltrate (FCSu 10 % v/v), 0.5 mM methyl-β-cyclodextrin (MβCD), or increasing concentrations of cholesterol-sulfate (Chol-SO_4_). After washing, sperm samples were incubated with various fluorescent dyes to assess cellular parameters: (**a**) anti-8-OHdG FITC-conjugated antibody (1:1000) for DNA oxidation and Sytox Blue (0.2 μM) for viability; (**b**) MitoSOX (2 μM) for mitochondrial superoxide (O_2_^•-^) production and Sytox Blue (0.2 μM) for viability; (**c**) JC-1 (2 μM) for mitochondrial membrane potential and Sytox Blue (0.2 μM); and (**d**) BODIPY C11 (5 μM) for lipid peroxidation and Sytox Blue (0.2 μM). Both live (Q3) and dead (Q2) cells were analyzed. Data are presented as the ratio of red/green fluorescence intensity in (**c**), which indicates membrane depolarization when the ratio decreases, and as the percentage of cells for (**a**) positive for 8-OHdG, (**b**) positive for production of O_2_^•-^, and (**d**) positive for lipid peroxidation. Results represent sperm samples from 4 different healthy donors (n = 4). Statistical analysis was performed using ANOVA with Tukey's test: ∗p ≤ 0.05, ∗∗p ≤ 0.01, and ∗∗∗p ≤ 0.001.

**Supplementary Fig. S4:**
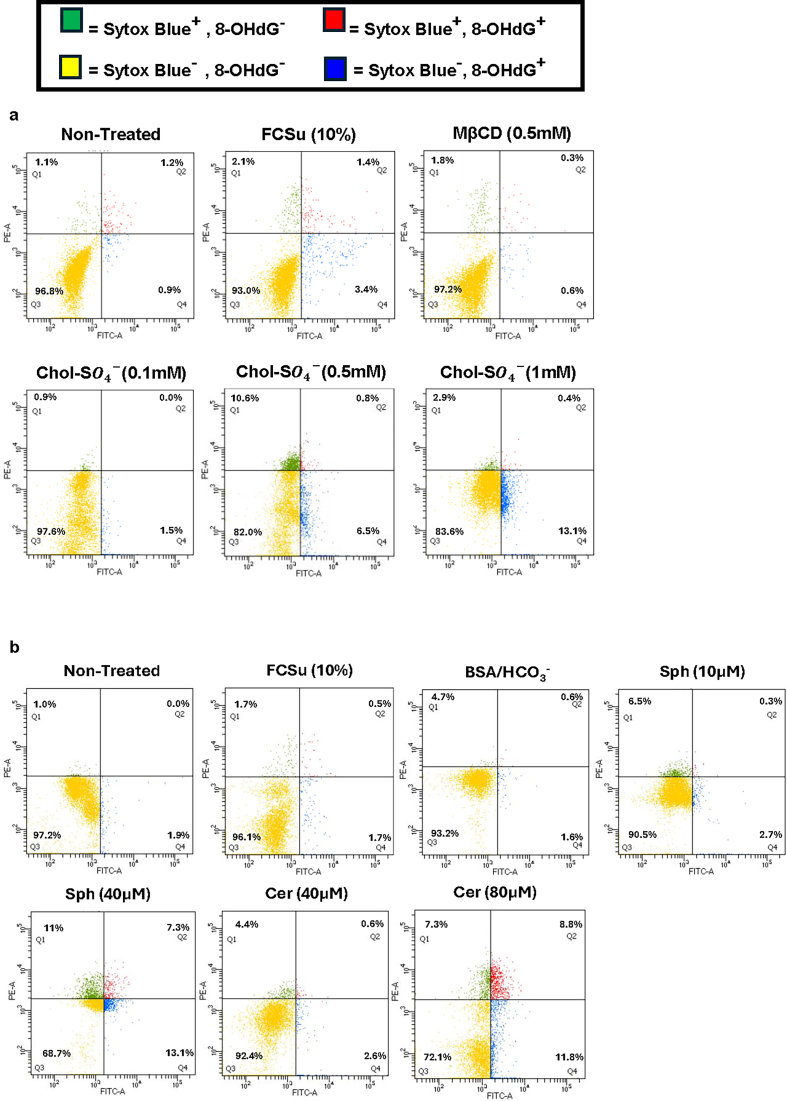
High doses of sphingolipids and cholesterol sulfate (Chol-SO_4_) on DNA **oxidation.** Representative dot plots (**a**) from the experiment shown in Fig. 5a and (**b**) from Fig. 6a illustrate DNA oxidation in spermatozoa after 3.5 h of incubation at 37 °C. The sperm samples were treated with fetal cord serum ultrafiltrate (FCSu), bovine serum albumin (BSA), (**a**) methyl β-cyclodextrin (MβCD) & Chol-SO_4_, and (**b**) sphingosine (Sph) and Ceramide (Cer). Live spermatozoa (Sytox Blue^−^) were classified as either 8-OHdG^+^ (blue, Q4) or 8-OHdG^−^ (yellow, Q3) based on DNA oxidation. Q1 and Q2 represent dead spermatozoa (Sytox Blue^+^) and 8-OHdG^−^ (green, Q1) or 8-OHdG^+^ (red, Q2) spermatozoa, respectively. The percentages of spermatozoa in each quadrant were quantified.

**Supplementary Fig. S5:**
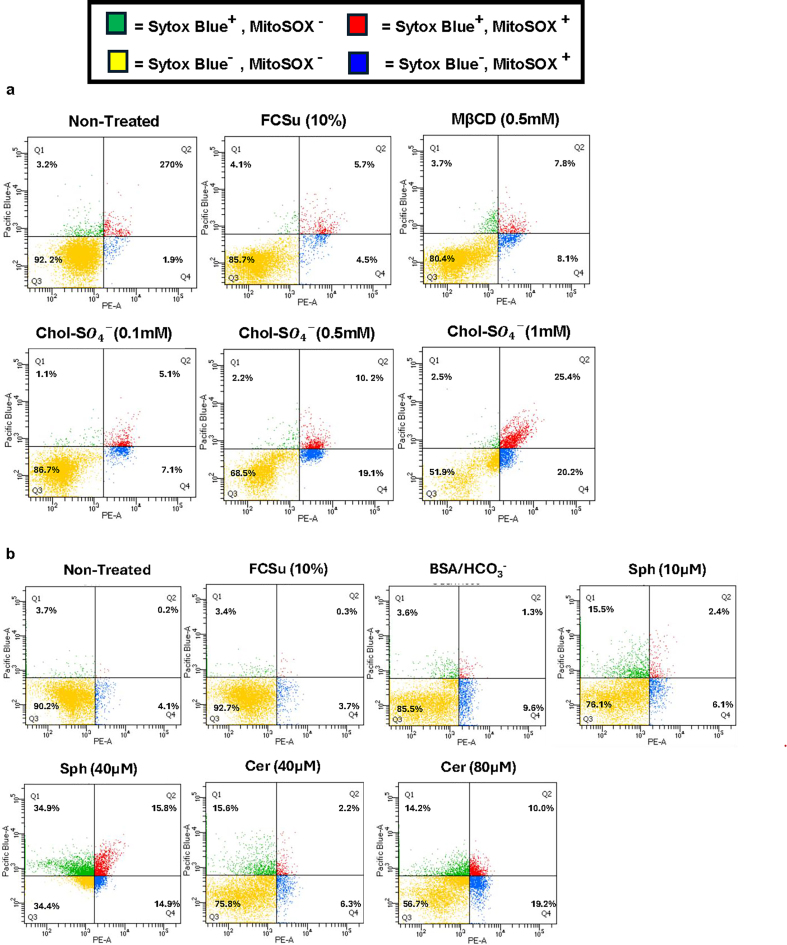
High doses of sphingolipids and cholesterol sulfate (Chol-SO_4_) affect mitochondrial membrane poten**tial (MMP)**. Representative dot plots (**a**) from the experiment in Fig. 5c and (**b**) from the experiment in Fig. 6c demonstrate MMP in spermatozoa after 3.5 h of incubation. The sperm samples were treated with fetal cord serum ultrafiltrate (FCSu), bovine serum albumin (BSA), (**a**) methyl β-cyclodextrin (MβCD) & Chol-SO_4_, and (**b**) sphingosine (Sph) and Ceramide (Cer). Live spermatozoa (Sytox Blue^−^) were categorized as either JC-1^+^ (high MMP, JC-1 forms J-aggregates in the mitochondria) (blue, Q4) or JC-1^−^ (low MMP, JC-1 remains in its monomeric form and emits green) (yellow, Q3) based on MMP. Q1 and Q2 represent dead spermatozoa (Sytox Blue^+^) and JC-1^−^ (Q1, green) or JC-1^+^ (Q2, red) sperm, respectively. The percentages of spermatozoa in each quadrant were quantified.

**Supplementary Fig. S6:**
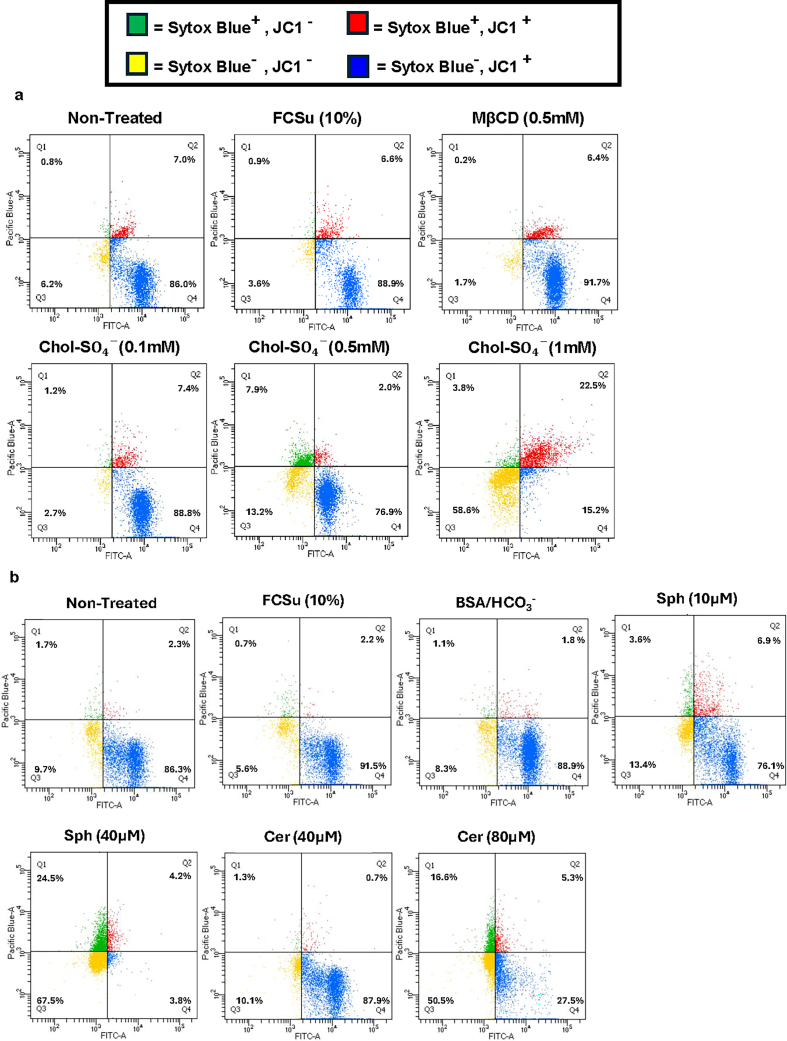
The effect of high doses of sphingolipids and cholesterol sulfate (Chol-SO_4_) on mitochondrial ROS **production**. Representative dot plots (**a**) from the experiment shown in Fig. 5b and (**b**) from Fig. 6b illustrate mitochondrial ROS production in spermatozoa after 3.5 h of incubation. The sperm samples were treated with fetal cord serum ultrafiltrate (FCSu), bovine serum albumin (BSA), (**a**) methyl β-cyclodextrin (MβCD) & Chol-SO_4_, and (**b**) sphingosine (Sph) and Ceramide (Cer). Live spermatozoa (Sytox Blue^−^) were classified as either MitoSOX^+^ (blue, Q4) or MitoSOX−(yellow, Q3) based on mitochondrial superoxide production. Quadrants Q1 and Q2 represent dead spermatozoa (Sytox Blue^+^) and MitoSOX^−^ (green, Q1) or MitoSOX^+^ (red, Q2) spermatozoa, respectively. The percentages of spermatozoa in each quadrant were quantified.

We studied increasing levels of Chol-SO_4_ on human sperm DNA oxidation. First, we report a dose-dependent increase in the percentage of spermatozoa with elevated levels of 8-OHdG only upon treatment with Chol-SO_4_ (Fig. 6a & Supplementary Fig. S4a), indicative of increased DNA damage. In the same experimental conditions, we also found that the 0.5 mM and 1 mM of Chol-SO_4_ led to a significant decrease in mitochondrial membrane potential (MMP) (Fig. 6c & Supplementary Fig. S6a), indicating a decrease in the electrochemical gradient across the inner mitochondrial membrane, which is essential for mitochondrial function, as well as an increase in mitochondrial O_2_^•-^ production (Fig. 6b & Supplementary Fig. S5a).

**Supplementary Fig. S7:**
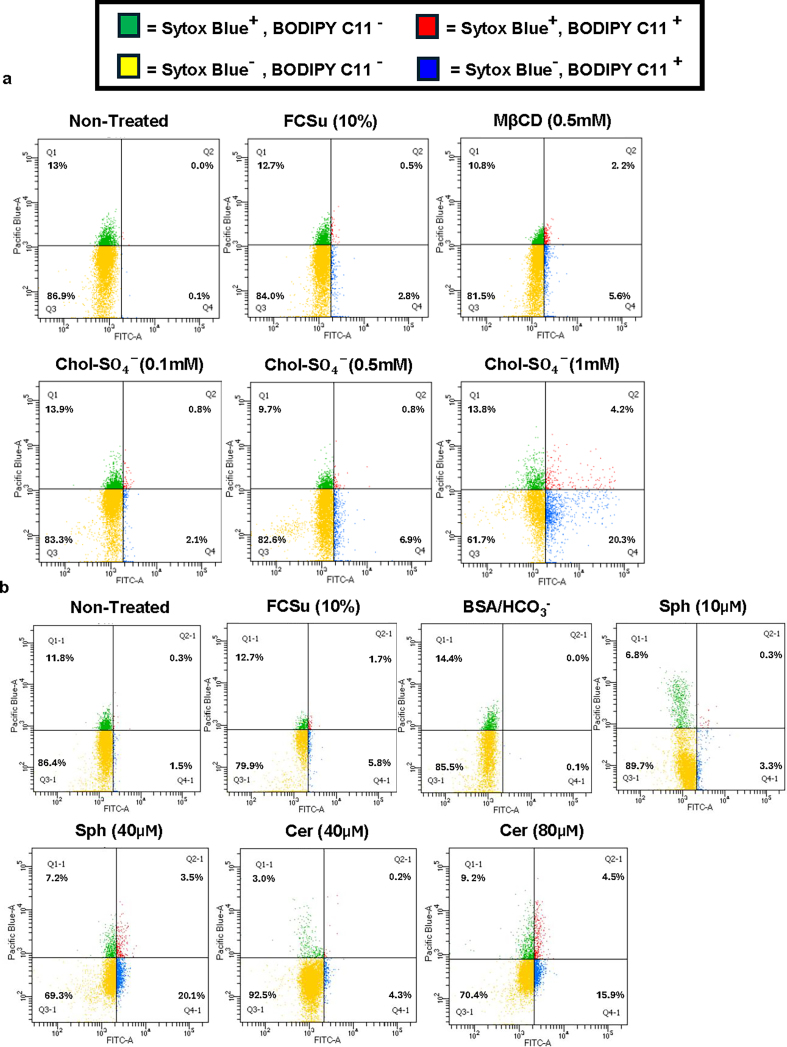
High doses of sphingolipids and cholesterol sulfate (Chol-SO4) affect lipid pe**roxidation**. Representative dot plots (**a**) from the experiment in Fig. 6d and (**b**) from the experiment in Fig. 7d demonstrate lipid peroxidation in spermatozoa after 3.5 h of incubation. The sperm samples were treated with fetal cord serum ultrafiltrate (FCSu), bovine serum albumin (BSA), (**a**) methyl β-cyclodextrin (MβCD) & Chol-SO_4_, and (**b**) sphingosine (Sph) and Ceramide (Cer). Live spermatozoa (Sytox Blue^−^) were categorized as either BODPY C11^+^ (blue, Q4) or BODPY C11^−^ (yellow, Q3) based on lipid peroxidation. Quadrants Q1 and Q2 represent dead spermatozoa (Sytox Blue^+^) and BODPY C11^−^ (green, Q1) or BODIPY C11^+^ (red, Q2) spermatozoa, respectively. The percentages of spermatozoa in each quadrant were quantified.

We found that 0.5 and 1 mM of Chol-SO_4_ significantly increased the percentage of cells exhibiting elevated lipid peroxidation, a marker of oxidative damage to polyunsaturated fatty acids in the cell membranes (Fig. 6d & Supplementary Fig. S7a).

### 3.7 Increasing concentrations of Sph and Cer promote excessive oxidative stress

**Fig. 7:**
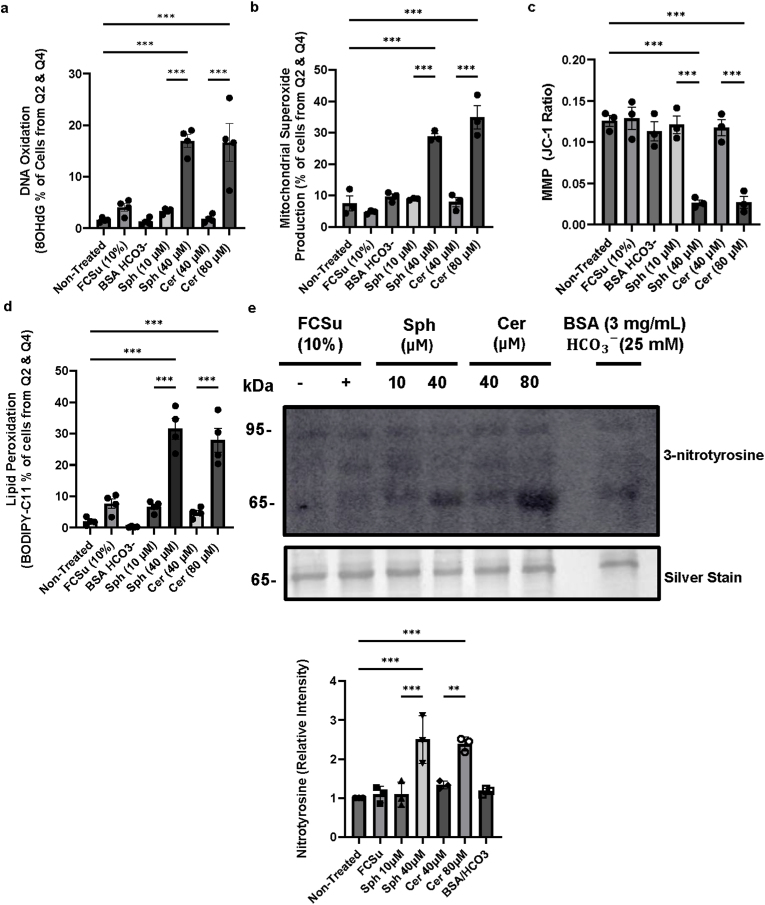
**High levels of Sphingosine and Ceramide invoke oxidative stress and damage**. Spermatozoa were incubated for 3.5 h at 37 °C with or without (Non-Treated) treatment with fetal cord serum ultrafiltrate (FCSu 10 % v/v), bovine serum albumin/bicarbonate (BSA/HCO_3_), 10 μM and 40 μM sphingosine (Sph), or 40 μM and 80 μM ceramide (Cer). After washing, sperm samples were incubated with the following probes: (**a**) anti-8-OHdG FITC-conjugated antibody (1:1000) for DNA oxidation and Sytox Blue (0.2 μM) for viability; (**b**) MitoSOX (2 μM) for mitochondrial superoxide (O_2_^•-^) production and Sytox Blue (0.2 μM); (**c**) JC-1 (2 μM) for mitochondrial membrane potential and Sytox Blue (0.2 μM); (**d**) BODIPY-C11 (5 μM) for lipid peroxidation and Sytox Blue (0.2 μM); and (**e**) immunoblotting with anti-3-nitrotyrosine (1:1000) for protein nitration. Flow cytometry analysis was performed to identify live (Q3) and dead (Q2) cells. Data are expressed as either the ratio of red/green fluorescence intensity in (**c**), indicating depolarization when the ratio decreases, or as the percentage of cells positive for 8-OHdG (**a**), O_2_^•−^production (**b**), and lipid peroxidation (**d**). Immunoblot analysis in (**e**) reflects the relative intensity of 3-nitrotyrosine, with each lane normalized to its silver-stain optical density for signal quantification. Results represent sperm samples from 4 different healthy donors (n = 4). Statistical analysis was performed using ANOVA with Tukey's test: ∗p ≤ 0.05, ∗∗p ≤ 0.01, and ∗∗∗p ≤ 0.001.

In the context of sphingolipids, we observed that 40 μM of Sph and 80 μM of Cer caused a significant increase in the percentage of cells exhibiting 8-OHdG, compared to their lower dose counterparts that promote capacitation [33] (Fig. 7a & Supplementary Fig. S4b). Higher doses of Sph and Cer also increase mitochondrial O_2_^•-^ production (Fig. 7b & Supplementary Fig. S5b) and reduce MMP (Fig. 7c & Supplementary Fig. S6b). In addition, we demonstrated that Sph at 40 μM and Cer at 80 μM also increase lipid peroxidation in spermatozoa (BODIPY C11^+^ cells) (Fig. 7d & Supplementary Fig. S7b).

Moreover, given the demonstrated ability of Sph at 10 μM and Cer at 40 μM to promote NO production [33], we evaluated the tyrosine nitration of sperm proteins when exposed to higher concentrations of the sphingolipid metabolites. We found that Sph at 40 μM and Cer at 80 μM promoted a higher intensity of 3-nitrotyrosine than their lower dose counterparts and FCSu-, MβCD-, and BSA-treated spermatozoa (Fig. 7e). Thus, the increase in NO production raises the possibility of enhanced peroxynitrite (ONOO^−^) formation, which could alter protein structure and interactions, potentially impacting their function and influencing sperm motility.

### 3.8 Preexposure of spermatozoa to elevated Chol-SO_4_, Sph, or Cer impairs their ability to capacitate

**Fig. 8:**
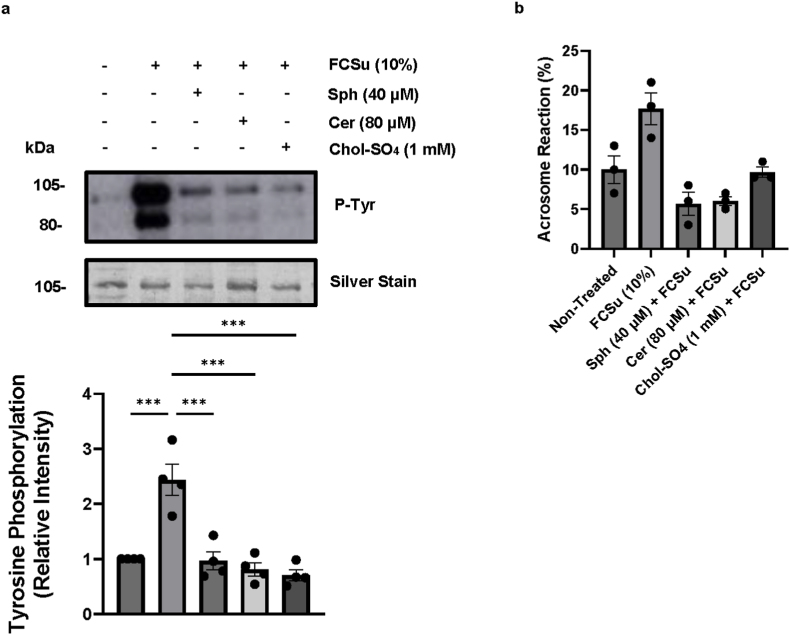
**Pre-treatment of sperm with high levels of sphingolipid and cholesterol impairs FCSu-induced capacitation.** Spermatozoa were first pre-treated with 40 μM sphingosine (Sph), 80 μM of Ceramide (Cer), or 1 mM cholesterol-sulfate (Chol-SO_4_) for 15 min at 37 °C. Then the samples were washed and incubated for 3.5 h at 37 °C, without any treatment (Non-Treated) or 10 %v/v Fetal cord serum ultrafiltrate (FCSu). Immunoblot analysis reveals a decrease in tyrosine phosphorylation (P-Tyr) in sperm samples pre-treated with high levels of sphingolipids or cholesterol. Each lane was normalized to its silver-stain optical density value for accurate signal quantification. Data represent sperm samples from 4 different healthy donors (n = 4). Statistical analysis was performed using ANOVA with Tukey's test: ∗p ≤ 0.05, ∗∗p ≤ 0.01, and ∗∗∗p ≤ 0.001.

To investigate whether dysregulated cholesterol and sphingolipid homeostasis, which impairs sperm capacitation, could be considered a cause of male factor infertility, we exposed spermatozoa to our pre-determined elevated lipid levels (1 mM Chol-SO_4_, 40 μM Sph, or 80 μM Cer) for 30 min at 37 °C. This approach was designed to mimic the lipid environment before and during ejaculation by pre-treating the spermatozoa *in vitro*. After the washing of the spermatozoa and incubating with FCSu in fresh BWW medium, spermatozoa pre-treated with Chol-SO_4_, Sph, or Cer did not show an increase in P-Tyr compared to spermatozoa treated with FCSu alone (Fig. 8a). Moreover, these pre-treated spermatozoa did not respond to progesterone and undergo AR (Fig. 8b).

## 4 Discussion

Our study presented novel insights into the role of lipid signalling, specifically cholesterol efflux and the crosstalk with sphingolipids, in regulating human sperm capacitation. A key finding of our research is that 0.5 mM MβCD induces human sperm capacitation that is reflected in cholesterol efflux, phosphorylation of tyrosine residues (P-Tyr) and PI3K (P-PI3K) (Fig. 1, Fig. 2a), hyperactivated motility and the progesterone-induced AR (Fig. 1b & c), endowing the sperm with fertilizing ability. This finding is significant because MβCD is commonly used to disrupt lipid rafts and induce cholesterol efflux [89]. Yet, its role in capacitation and the specific signalling pathways involved had not been fully elucidated.

Our study demonstrates that MβCD-mediated P-Tyr and P-PI3K activation is blocked by inhibiting S1P-mediated S1PR1/3 signaling (Fig. 1, Fig. 2a), highlighting a previously unrecognized mechanism in which cholesterol efflux and sphingolipid signaling interact and converge during capacitation. Furthermore, we discovered a novel interaction between MβCD-mediated cholesterol efflux and intracellular NO production during capacitation (Fig. 2, Fig. 3a). Given that our previous studies have shown sphingolipids to regulate NO production during capacitation [33], the findings presented above further demonstrate the convergence of MβCD and sphingolipid signalling through increased phosphorylation of PI3K, activation of AKT-substrate NOS, and subsequent NO production. This finding highlights the crucial role of lipid raft-mediated signalling in facilitating the biochemical and biophysical changes that happen during the human sperm capacitation process.

Although it has not been demonstrated whether cholesterol efflux or sphingolipid signalling comes before the other during capacitation, cholesterol efflux is an early event mediated by HDL and albumin found within the oviductal and follicular fluid [90]. Hence, cholesterol efflux can trigger capacitation through membrane remodelling, activating proteins involved in sphingolipid signalling. In macrophages, S1P acts as a positive regulator of cholesterol efflux by modulating the activity of ABCA1 and ABCG1 [91]. Moreover, Cer and SM can influence cholesterol retention in the plasma membrane [80]; hence, further research is required to determine the order of these events.

For the first time, we demonstrate that, in addition to increasing membrane fluidity [92] and facilitating interactions between previously segregated membrane proteins to initiate signalling, MβCD-mediated capacitation promotes O_2_^•−^ production at the level of the sperm plasma membrane. Notably, the addition of cell-impermeable SOD1 prevented capacitation, highlighting the critical role of extracellular O_2_^•−^ in this process (Fig. 3b and c). It is known that capacitation-associated O_2_^•−^ production occurs at the plasma membrane in species like human and bovine spermatozoa [65,[93], [94]]. Thus, we can suggest that the cholesterol efflux reconfigures the plasma membrane, allowing for the activation of the sperm oxidase in the early stages of capacitation. This O_2_^•−^ production triggers the activation of protein kinase R (PKR), which subsequently activates sphingolipid signalling by recruiting and activating SphK1, as previously shown [33]. Phosphorylated SphK1 (P-SphK1) converts Sph to S1P, which binds to S1PR1, activating the PI3K-AKT pathway and the production of NO. This cascade increases protein P-Tyr, which in turn promotes hyperactivated motility, facilitates zona pellucida recognition and binding, induces acrosomal exocytosis in response to the zona pellucida, and enables spermatozoa to respond to progesterone and undergo the acrosome reaction. These events collectively indicate that the sperm has successfully undergone capacitation. These findings highlight the intricate interaction between cholesterol and sphingolipids, emphasizing the role of cholesterol and associated machinery in maintaining low ROS levels during sperm capacitation.

A key innovation of our study is its investigation of nSMase inhibition on cholesterol efflux mediated by MβCD, providing new insights into the regulatory mechanisms of lipid metabolism in human spermatozoa. Previous research has demonstrated that SM regulates the sperm acrosome reaction through cholesterol depletion [88]. However, our work expands on this by showing that nSMase inhibition with 10 μM of 3-OMS impairs both FCSu- and MβCD-induced P-Tyr (Fig. 4a and b), most likely through the retention of cholesterol (Fig. 4c). Despite demonstrating that the retention of cholesterol coincides with a decline in P-Tyr upon nSMase inhibition with 10 μM of 3-OMS, further studies are needed to validate this hypothesis by directly inhibiting cholesterol efflux and assessing its impact on P-Tyr levels.

SM retains cholesterol within the cell membrane through several mechanisms. One way it does this is by forming lipid rafts, creating an environment in which ordered, less fluid regions are favoured by cholesterol due to its ability to integrate into these tightly packed domains. The formation of lipid rafts slows the extraction of raft-associated cholesterol compared to non-raft cholesterol, as raft-based cholesterol is tightly bound to SM and other lipids, which helps stabilize it within the membrane [95].

Additionally, the structure of SM, which features a long saturated fatty acid chain and a polar head group, allows it to interact with cholesterol through hydrogen bonds and van der Waals forces. These interactions further anchor cholesterol to the membrane, preventing dissociation [96]. By modulating membrane fluidity and facilitating the formation of stable, ordered domains, SM ensures that cholesterol remains well-integrated within the membrane, maintaining membrane integrity and function. In spermatozoa, SM is converted by SMase to Cer, which is then converted by ceramidase to Sph, promoting the cholesterol efflux necessary to trigger the early events of capacitation. This highlights the link between sphingolipids and cholesterol signalling within lipid rafts during capacitation.

In addition to the findings mentioned above, our work introduces the novel concept of lipid metabolism dysregulation that impairs sperm capacitation. Previous studies have linked high cholesterol and sphingolipid concentrations to reduced male fecundity [58,97] and altered sperm function [20,24,25]. Moreover, infertile males have presented with elevated levels of ceramide (Cer) in their seminal plasma [98]. In a hamster model of insulin resistance, a high-fat diet increases circulating sphingolipid metabolites, particularly Cer, along with elevated plasma triglycerides, cholesterol, and VLDL-TG levels [[99], [100], [101],^102^]. However, the regulatory role of sphingolipids and cholesterol in dyslipidemia associated with male infertility remains poorly understood. We provide compelling evidence that exposure of spermatozoa to increasing concentrations of Chol-SO_4_, Sph, and Cer *in vitro* impairs key aspects of human sperm function, including capacitation (Fig. 5), motility (Supplementary Figs. S2a and S3a), and viability (Supplementary Figs. S2b and S3b). These findings underscore lipid dysregulation, a previously underexplored but potentially significant factor in male infertility.

Moreover, the diet-induced elevation of cholesterol and sphingolipids, particularly Cer, increased oxidative stress in different cell types [[99], [100], [101], [102], [103], [104]]. Elevated oxidative stress has been shown to negatively impact sperm motility and capacitation, primarily by promoting redox-dependent protein modifications. These modifications, such as oxidation and S-nitrosylation, can disrupt key proteins in sperm. According to Morielli and O'Flaherty (2015), oxidative stress impairs sperm function by inducing redox modifications, including the oxidation of thiol groups on proteins, leading to alterations in their structure and function [105]. These modifications can impair the sperm's ability to undergo capacitation and reduce motility, limiting fertilization potential. Lefievre et al. (2007) also highlighted that human spermatozoa are highly susceptible to protein S-nitrosylation, a redox-dependent modification mediated by the accumulation of ONOO^−^ [106]. S-nitrosylation can modulate the activity of various sperm proteins, influencing motility and capacitation. For example, S-nitrosylation of key enzymes involved in sperm energy production and motility may reduce their function, impairing sperm movement and fertilization capacity. In Fig. 7e, 40 μM of Sph and 80 μM of Cer increased 3-nitrotyrosine in protein bands of 60–70 kDa. Potential candidates include heat shock protein 70 (HSP70), which is tyrosine phosphorylated during human sperm capacitation, can be modified by nitrosylation, and may have ties to motility [106,107]. Together, these studies underscore the critical role of oxidative stress in male fertility, as the accumulation of redox protein modifications can significantly compromise sperm function and impair reproductive outcomes.

Ultimately, our study demonstrates that elevated levels of Chol-SO_4_, Sph, and Cer can promote oxidative stress and damage that can concur with the impairment of sperm function, including capacitation, motility, and acrosome reaction. This is evidenced by increased sperm DNA oxidation (Fig. 6, Fig. 7a), reduced mitochondrial membrane potential (MMP) (Fig. 6, Fig. 7c), heightened mitochondrial O_2_^•-^ production (Fig. 6, Fig. 7b), lipid peroxidation (Fig. 6, Fig. 7d), and tyrosine nitrosylation (Fig. 7e). Moreover, we cannot exclude the contribution of dysregulated NADPH oxidase 5 (NOX5). Its excessive production of reactive oxygen and nitrogen species (RONS) may lead to oxidative stress and subsequent damage to DNA and plasma membrane integrity [105]. Hence, this merits future research on the matter. The elevated oxidative stress compromises sperm membrane integrity, increasing its permeability and generating 4-hydroxy-2-nonenal (4-HNE). These reactive products can damage proteins and other cellular components, further disrupting sperm function [108].

**Fig. 9:**
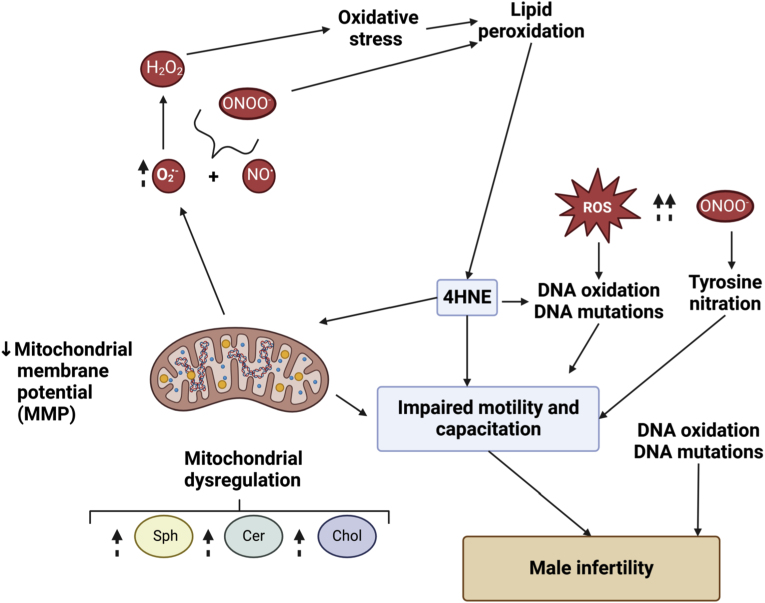
**Dysregulation of sphingolipid rheostat and cholesterol homeostasis promotes oxidative stress in human spermatozoa, impacting male fertility.** Elevated levels of Sphingosine (Sph), Ceramide (Cer), and cholesterol-sulfate (Chol-SO_4_) result in mitochondrial dysregulation and leakage, causing an uptick in superoxide production. The formation of hydrogen peroxide (H_2_O_2_) and peroxynitrite (ONOO^−^) can promote oxidative stress and lipid peroxidation. The release of cytotoxic byproduct 4-HNE can further exacerbate the loss of mitochondrial function, impair motility, cause DNA damage (DNA oxidation and mutations), and impair sperm capacitation. Moreover, an increase in ROS generation can directly increase DNA oxidation, impairing sperm function. More specifically, a high level of ONOO^−^ can lead to the tyrosine-nitration of protein, which is important for sperm motility and capacitation. Altogether, dysregulated sphingolipid and cholesterol homeostasis can result in male infertility. This figure was generated using BioRender.

These findings highlight the relationship between high lipid levels and the promotion of damaging oxidative stress in human spermatozoa. Our study offers new insights into the role of sphingolipid regulation and cholesterol homeostasis dysregulation in the incomplete or altered fertilizing ability of spermatozoa. We are the first to establish that excess sphingolipids and Chol-SO_4_ alter the sperm's ability to undergo capacitation, emphasizing the delicate balance of lipid metabolism required for optimal sperm function (Fig. 9).

One notable limitation of the current study is the lack of semen samples from patients with dyslipidemia, which were not available for analysis. As a result, we utilized healthy semen samples from male volunteers. Moving forward, it is essential to obtain clinical samples from patients with dyslipidemia to obtain concrete values of sphingolipids and cholesterol in the sperm and seminal fluid compared to healthy semen samples from male volunteers. Moreover, this will provide a better understanding of the specific effects of this condition on semen quality and the altered fertilizing ability of the spermatozoa. Such studies would provide more accurate insights and contribute to a deeper understanding of how dyslipidemia may influence male fertility.

In summary, our research makes several novel contributions to the field of male reproductive biology. Specifically, it highlights how lipid raft components, sphingolipids, and cholesterol integrate into complex pathways that regulate sperm capacitation. These findings lay the foundation for future investigations into lipid-mediated signalling in sperm function. Additionally, we present new evidence on how lipid dysregulation disrupts capacitation and sperm function, providing a potential mechanism for male infertility linked to dyslipidemia. The data presented offers valuable insights into the signalling cascades that could influence male fertility, bridging the gap between basic research and translational medicine.

## Funding

This study was supported by the 10.13039/501100007202Canadian Institutes of Health Research (CIHR) under grant numbers PJT-165962 and PJT-191704, awarded to Cristian O'Flaherty. Additionally, Steven Serafini received a Research Institute-MUHC Desjardins Studentship.

## CRediT authorship contribution statement

**Steven Serafini:** Writing – review & editing, Writing – original draft, Visualization, Validation, Methodology, Investigation, Formal analysis, Data curation, Conceptualization. **Cristian O'Flaherty:** Writing – review & editing, Writing – original draft, Validation, Supervision, Resources, Project administration, Methodology, Investigation, Funding acquisition, Formal analysis, Data curation, Conceptualization.

## Declaration of competing interest

None.

## Data Availability

Data will be made available on request.

## References

[1] Collins M.E. (2019). The impact of infertility on daily occupations and roles. J. Reproduction Infertil..

[2] Cox C. (2022). Infertility prevalence and the methods of estimation from 1990 to 2021: a systematic review and meta-analysis. Human Reproduction Open.

[3] Singh K., Jaiswal D. (2011). Human male infertility: a complex multifactorial phenotype. Reprod. Sci..

[4] Longo V. (2019). Ambient Assisted Living: Italian Forum 2018 9.

[5] Aitken R.J. (2013). Falling sperm counts twenty years on: where are we now?. Asian J. Androl..

[6] Sahu A., Pajai S. (2023). The impact of obesity on reproductive health and pregnancy outcomes. Cureus.

[7] Davidson L.M. (2015). Deleterious effects of obesity upon the hormonal and molecular mechanisms controlling spermatogenesis and male fertility. Hum. Fertil..

[8] Blüher M. (2013). Adipose tissue dysfunction contributes to obesity related metabolic diseases. Best Pract. Res. Clin. Endocrinol. Metabol..

[9] Klop B., Elte J.W.F., Castro Cabezas M. (2013). Dyslipidemia in obesity: mechanisms and potential targets. Nutrients.

[10] Hamad Zubi Z.B., Hamad Alfarisi H.A. (2021). Hyperlipidemia and male infertility. Egyptian Journal of Basic and Applied Sciences.

[11] Miller M. (2009). Dyslipidemia and cardiovascular risk: the importance of early prevention. QJM: Int. J. Med..

[12] Brunham L.R., Lonn E., Mehta S.R. (2024). Dyslipidemia and the current state of cardiovascular disease: epidemiology, risk factors, and effect of lipid lowering. Can. J. Cardiol..

[13] Sowers J.R. (1992). Insulin resistance, hyperinsulinemia, dyslipidemia, hypertension, and accelerated atherosclerosis. J. Clin. Pharmacol..

[14] Bloomgarden Z.T. (2007). Insulin resistance, dyslipidemia, and cardiovascular disease. Diabetes Care.

[15] Koene R.J. (2016). Shared risk factors in cardiovascular disease and cancer. Circulation.

[16] Ferramosca A. (2016). A high‐fat diet negatively affects rat sperm mitochondrial respiration. Andrology.

[17] Jia Y.-F. (2018). Obesity impairs male fertility through long-term effects on spermatogenesis. BMC Urol..

[18] Li C. (2015). Endoplasmic reticulum stress promotes the apoptosis of testicular germ cells in hyperlipidemic rats. Zhonghua nan ke xue= National Journal of Andrology.

[19] Bataineh H.N., Nusier M.K. (2005). Effect of cholesterol diet on reproductive function in male albino rats. Saudi Med. J..

[20] Yamamoto Y. (1999). Effects of hypercholesterolaemia on Leydig and Sertoli cell secretory function and the overall sperm fertilizing capacity in the rabbit. Hum. Reprod..

[21] Ashrafi H. (2013). The effect of quince leaf (Cydonia oblonga Miller) decoction on testes in hypercholesterolemic rabbits: a pilot study. Afr. J. Tradit., Complementary Altern. Med..

[22] Shalaby M., El Zorba H., Kamel G.M. (2004). Effect of α-tocopherol and simvastatin on male fertility in hypercholesterolemic rats. Pharmacol. Res..

[23] Bashandy A.S. (2007). Effect of fixed oil of Nigella sativa on male fertility in normal and hyperlipidemic rats. Int. J. Pharmacol..

[24] Saez Lancellotti T.E. (2010). Hypercholesterolemia impaired sperm functionality in rabbits. PLoS One.

[25] Saez Lancellotti T.E. (2013). Semen quality and sperm function loss by hypercholesterolemic diet was recovered by addition of olive oil to diet in rabbit. PLoS One.

[26] Mu Y. (2016). Curcumin ameliorates high-fat diet-induced spermatogenesis dysfunction. Mol. Med. Rep..

[27] Mu Y. (2017). Diet-induced obesity impairs spermatogenesis: a potential role for autophagy. Sci. Rep..

[28] Chen X., Gong L., Xu J. (2012). 2012 International Conference on Biomedical Engineering and Biotechnology.

[29] Zhang K. (2012). Melatonin prevents testicular damage in hyperlipidaemic mice. Andrologia.

[30] Pushpendra A., Jain G. (2015). Hyper-lipidemia and male fertility: a critical review of literature. Andrology (Zanetti, #750).

[31] Aziz N. (2004). Novel association between sperm reactive oxygen species production, sperm morphological defects, and the sperm deformity index. Fertil. Steril..

[32] De Jonge C. (2017). Biological basis for human capacitation—revisited. Hum. Reprod. Update.

[33] Serafini S., O'Flaherty C. (2025). Sphingolipids modulate redox signalling during human sperm capacitation. Hum. Reprod..

[34] Visconti P.E. (1999). Cholesterol efflux-mediated signal transduction in mammalian sperm: cholesterol release signals an increase in protein tyrosine phosphorylation during mouse sperm capacitation. Dev. Biol..

[35] Travis A.J., Kopf G.S. (2002). The role of cholesterol efflux in regulating the fertilization potential of mammalian spermatozoa. J. Clin. Investig..

[36] Shadan S. (2004). Cholesterol efflux alters lipid raft stability and distribution during capacitation of boar Spermatozoa1. Biol. Reprod..

[37] Ehrenwald E., Foote R.H., Parks J.E. (1990). Bovine oviductal fluid components and their potential role in sperm cholesterol efflux. Mol. Reprod. Dev..

[38] Furse S. (2022). Relative abundance of lipid metabolites in spermatozoa across three compartments. Int. J. Mol. Sci..

[39] Boslem E., Meikle P.J., Biden T.J. (2012). Roles of Ceramide and sphingolipids in pancreatic β-cell function and dysfunction. Islets.

[40] Hannun Y.A., Obeid L.M. (2008). Principles of bioactive lipid signalling: lessons from sphingolipids. Nat. Rev. Mol. Cell Biol..

[41] Newton J. (2015). Revisiting the sphingolipid rheostat: evolving concepts in cancer therapy. Exp. Cell Res..

[42] Watterson K.R., Ratz P.H., Spiegel S. (2005). The role of sphingosine-1-phosphate in smooth muscle contraction. Cell. Signal..

[43] Józefczuk E., Guzik T., Siedlinski M. (2020). Significance of sphingosine-1-phosphate in cardiovascular physiology and pathology. Pharmacol. Res..

[44] Rohrhofer J. (2021). The impact of dietary sphingolipids on intestinal microbiota and gastrointestinal immune homeostasis. Front. Immunol..

[45] Czubowicz K. (2019). The role of Ceramide and sphingosine-1-phosphate in Alzheimer's disease and other neurodegenerative disorders. Mol. Neurobiol..

[46] Hait N.C., Maiti A. (2017). The role of sphingosine‐1‐phosphate and ceramide‐1‐phosphate in inflammation and cancer. Mediat. Inflamm..

[47] Zhu C. (2023). Insights into the roles and pathomechanisms of Ceramide and sphigosine-1-phosphate in nonalcoholic fatty liver disease. Int. J. Biol. Sci..

[48] Spiegel S., Merrill A.H. (1996). Sphingolipid metabolism and cell growth regulation. FASEB J..

[49] Hannun Y.A., Luberto C., Argraves K.M. (2001). Enzymes of sphingolipid metabolism: from modular to integrative signaling. Biochemistry.

[50] Borodzicz-Jażdżyk S. (2022). Sphingolipid metabolism and signaling in cardiovascular diseases. Frontiers in cardiovascular medicine.

[51] Kolter T., Sandhoff K. (2006). Sphingolipid metabolism diseases. Biochim. Biophys. Acta Biomembr..

[52] Piccinini M. (2010). Deregulated sphingolipid metabolism and membrane organization in neurodegenerative disorders. Mol. Neurobiol..

[53] Vaquer C.C. (2020). Ceramide induces a multicomponent intracellular calcium increase triggering the acrosome secretion in human sperm. Biochim. Biophys. Acta Mol. Cell Res..

[54] Wang D., Tang Y., Wang Z. (2023). Role of sphingolipid metabolites in the homeostasis of steroid hormones and the maintenance of testicular functions. Front. Endocrinol..

[55] Suomalainen L. (2003). Sphingosine-1-phosphate in inhibition of male germ cell apoptosis in the human testis. J. Clin. Endocrinol. Metabol..

[56] Li X. (2020). Sphingomyelin synthase 2 participate in the regulation of sperm motility and apoptosis. Molecules.

[57] Suhaiman L. (2010). Sphingosine 1-phosphate and sphingosine kinase are involved in a novel signaling pathway leading to acrosomal exocytosis. J. Biol. Chem..

[58] Grizard G. (1995). Cholesterol, phospholipids and markers of the function of the accessory sex glands in the semen of men with hypercholesterolaemia. Int. J. Androl..

[59] Cross N.L. (1996). Human seminal plasma prevents sperm from becoming acrosomally responsive to the agonist, progesterone: cholesterol is the major inhibitor. Biol. Reprod..

[60] Van Gestel R. (2005). Capacitation-dependent concentration of lipid rafts in the apical ridge head area of porcine sperm cells. Mol. Hum. Reprod..

[61] Cross N.L. (1998). Role of cholesterol in sperm capacitation. Biol. Reprod..

[62] Pitha J. (1988). Drug solubilizers to aid pharmacologists: amorphous cyclodextrin derivatives. Life Sci..

[63] de Lamirande E., Lamothe G. (2009). Reactive oxygen-induced reactive oxygen formation during human sperm capacitation. Free Radic. Biol. Med..

[64] Biggers J., Whitten W., Whittingham D. (1971). The Culture of Mouse Embryos in Vitro.

[65] de Lamirande E., Cagnon C. (1993). Human sperm hyperactivation and capacitation as parts of an oxidative process. Free Radic. Biol. Med..

[66] de Lamirande E. (1997). Reactive oxygen species and sperm physiology. Rev. Reprod..

[67] Leclerc P., De Lamirande E., Gagnon C. (1997). Regulation of protein-tyrosine phosphorylation and human sperm capacitation by reactive oxygen derivatives. Free Radic. Biol. Med..

[68] Leclerc P., de Lamirande E., Gagnon C. (1996). Cyclic adenosine 3′, 5′ monophosphate-dependent regulation of protein tyrosine phosphorylation in relation to human sperm capacitation and motility. Biol. Reprod..

[69] Ramu S., Jeyendran R.S. (2013). The hypo-osmotic swelling test for evaluation of sperm membrane integrity. Spermatogenesis: Methods and protocols.

[70] Organization W.H. (2021).

[71] Mortimer D., Mortimer S.T., Carrell D., Aston K. (2013). Spermatogenesis. Methods in Molecular Biology.

[72] Baldi E. (1991). Intracellular calcium accumulation and responsiveness to progesterone in capacitating human spermatozoa. J. Androl..

[73] de Lamirande E., Gagnon C. (1995). Capacitation-associated production of superoxide anion by human spermatozoa. Free Radic. Biol. Med..

[74] Fernandez M.C., O'Flaherty C. (2018). Peroxiredoxin 6 is the primary antioxidant enzyme for the maintenance of viability and DNA integrity in human spermatozoa. Hum. Reprod..

[75] Lee D. (2017). Peroxiredoxins prevent oxidative stress during human sperm capacitation. MHR: Basic science of reproductive medicine.

[76] Moawad A.R. (2017). Deficiency of peroxiredoxin 6 or inhibition of its phospholipase A2 activity impair the in vitro sperm fertilizing competence in mice. Sci. Rep..

[77] Koppers A.J. (2008). Significance of mitochondrial reactive oxygen species in the generation of oxidative stress in spermatozoa. J. Clin. Endocrinol. Metabol..

[78] Luconi M. (2004). Increased phosphorylation of AKAP by inhibition of phosphatidylinositol 3-kinase enhances human sperm motility through tail recruitment of protein kinase A. J. Cell Sci..

[79] Herrero M. (1996). Localization by indirect immunofluorescence of nitric oxide synthase in mouse and human spermatozoa. Reprod. Fertil. Dev..

[80] Nixon B. (2011). Proteomic and functional analysis of human sperm detergent resistant membranes. J. Cell. Physiol..

[81] O'Flaherty C., de Lamirande E., Gagnon C. (2006). Positive role of reactive oxygen species in mammalian sperm capacitation: triggering and modulation of phosphorylation events. Free Radic. Biol. Med..

[82] O'Flaherty C., de Lamirande E., Gagnon C. (2006). Reactive oxygen species modulate independent protein phosphorylation pathways during human sperm capacitation. Free Radic. Biol. Med..

[83] de Lamirande E., Leclerc P., Gagnon C. (1997). Capacitation as a regulatory event that primes spermatozoa for the acrosome reaction and fertilization. Mol. Hum. Reprod..

[84] Herrero M.B., de Lamirande E., Gagnon C. (1999). Nitric oxide regulates human sperm capacitation and protein-tyrosine phosphorylation in vitro. Biol. Reprod..

[85] Olds-Clarke P. (2003). Unresolved issues in mammalian fertilization. Int. Rev. Cytol..

[86] Thundathil J., de Lamirande E., Gagnon C. (2003). Nitric oxide regulates the phosphorylation of the threonine-glutamine-tyrosine motif in proteins of human spermatozoa during capacitation. Biol. Reprod..

[87] de Lamirande E., O'Flaherty C. (2012). Studies on Men's Health and Fertility.

[88] Cross N.L. (2000). Sphingomyelin modulates capacitation of human sperm in vitro. Biol. Reprod..

[89] Zidovetzki R., Levitan I. (2007). Use of cyclodextrins to manipulate plasma membrane cholesterol content: evidence, misconceptions and control strategies. Biochim. Biophys. Acta Biomembr..

[90] Bailey J.L. (2010). Factors regulating sperm capacitation. Syst. Biol. Reprod. Med..

[91] Vaidya Mithila (2019). Regulation of ABCA1-mediated cholesterol efflux by sphingosine-1-phosphate signaling in macrophages. JLR (J. Lipid Res.).

[92] Companyó M., Iborra A., Villaverde J., Martínez P., Morros A. (2007). Membrane fluidity changes in goat sperm induced by cholesterol depletion using beta-cyclodextrin. Biochim. Biophys. Acta Biomembr..

[93] O'Flaherty C., Beorlegui N., Beconi M. (2003). Participation of superoxide anion in the capacitation of cryopreserved bovine sperm. Int. J. Androl..

[94] O'Flaherty C., Beorlegui N., Beconi M. (1999). Reactive oxygen species requirements for bovine sperm capacitation and acrosome reaction. Theriogenology.

[95] Simons K., Ikonen E. (2000). How cells handle cholesterol. Science.

[96] Slotte J.P. (2016). The importance of hydrogen bonding in sphingomyelin's membrane interactions with co-lipids. Biochim. Biophys. Acta Biomembr..

[97] Wei M. (1994). Total cholesterol and high density lipoprotein cholesterol as important predictors of erectile dysfunction. Am. J. Epidemiol..

[98] Correnti Serena (2023). Seminal plasma untargeted metabolomic and lipidomic profiling for the identification of a novel panel of biomarkers and therapeutic targets related to male infertility. Front. Pharmacol..

[99] Yeung C. (2001). Changes of the major sperm maturation-associated epididymal protein HE5 (CD52) on human ejaculated spermatozoa during incubation in capacitation conditions. Mol. Hum. Reprod..

[100] Dekker M.J. (2013). Inhibition of sphingolipid synthesis improves dyslipidemia in the diet-induced hamster model of insulin resistance: evidence for the role of Sphingosine and sphinganine in hepatic VLDL-apoB100 overproduction. Atherosclerosis.

[101] Stranahan A.M. (2011). Diet‐induced elevations in serum cholesterol are associated with alterations in hippocampal lipid metabolism and increased oxidative stress. J. Neurochem..

[102] Clement A.B. (2009). Adaptation of neuronal cells to chronic oxidative stress is associated with altered cholesterol and sphingolipid homeostasis and lysosomal function. J. Neurochem..

[103] Cutler R.G. (2002). Evidence that accumulation of ceramides and cholesterol esters mediates oxidative stress–induced death of motor neurons in amyotrophic lateral sclerosis. Ann. Neurol.: Official Journal of the American Neurological Association and the Child Neurology Society.

[104] Cutler R.G. (2004). Involvement of oxidative stress-induced abnormalities in ceramide and cholesterol metabolism in brain aging and Alzheimer's disease. Proc. Natl. Acad. Sci..

[105] Morielli T., O'Flaherty C. (2015). Oxidative stress impairs function and increases redox protein modifications in human spermatozoa. Reproduction.

[106] Lefièvre L. (2007). Human spermatozoa contain multiple targets for protein S‐nitrosylation: an alternative mechanism of the modulation of sperm function by nitric oxide?. Proteomics.

[107] Grassi S. (2022). Targeted analysis of HSP70 isoforms in human spermatozoa in the context of capacitation and motility. Int. J. Med. Sci..

[108] Baker Mark A. (2015). Defining the mechanisms by which the reactive oxygen species by-product, 4-hydroxynonenal, affects human sperm cell function. Biol. Reprod..

